# Residual Lung Cancer After Incomplete Microwave Ablation Exhibits cGAS–STING–ZEB1-Driven Malignant Progression

**DOI:** 10.3390/cancers18162594

**Published:** 2026-08-12

**Authors:** Chuanfei Zhan, Yuanyuan Zhai, Tianming Chen, Xiaokang Shen, Zi Wang, Shuliang Ma, Shilin Chen

**Affiliations:** 1 Department of Critical Care Unit, Jiangsu Cancer Hospital & Jiangsu Institute of Cancer Research & The Affiliated Cancer Hospital of Nanjing Medical University, Nanjing 210000, China; zhanchuanfei@163.com (C.Z.); mashuliang@163.com (S.M.); 2 Department of Thoracic Surgery, Jiangsu Cancer Hospital & Jiangsu Institute of Cancer Research & The Affiliated Cancer Hospital of Nanjing Medical University, Nanjing 210000, China; zhaiyuanyuan@stu.syphu.edu.cn (Y.Z.); wangzi@stu.njmu.edu.cn (Z.W.); 3 Department of General Surgery, Nanjing Drum Tower Hospital, Drum Tower Clinical Medical College, Nanjing Medical University, The Affiliated Hospital of Nanjing University Medical School, Nanjing 210009, China; tianmingchen@stu.njmu.edu.cn; 4 Department of Thoracic and Cardiovascular Surgery, Nanjing First Hospital, Nanjing Medical University, Nanjing 210006, China; shenxiaokang@njmu.edu.cn

**Keywords:** microwave ablation, non-small cell lung carcinoma, cGAS-STING signaling pathway, ZEB1, mtDNA, autophagy, tumor recurrence

## Abstract

Background: Incomplete microwave ablation (iMWA) of lung cancer often leads to rapid recurrence and metastasis, yet the underlying mechanisms remain unclear. This study explored whether iMWA promotes tumor progression by activating the cyclic GMP–AMP synthase–stimulator of interferon genes (cGAS–STING) signaling pathway and its downstream effector ZEB1 in tumor cells. Materials and Methods: An in vivo iMWA model was established in nude mice bearing H1650 lung tumors, and an in vitro sublethal heat treatment model was used to mimic incomplete ablation. Transcriptomic profiling, molecular assays and functional analyses assessed cellular behavior and signaling activity changes post-iMWA; genetic and pharmacologic interventions modulated STING signaling and autophagy. Results: Post-iMWA residual cells exhibited enhanced proliferation and invasion. Thermal injury induced necrosis and inhibited mitophagy, causing cytosolic mtDNA accumulation that activated the intrinsic cGAS–STING pathway. This upregulation of ZEB1 drove epithelial–mesenchymal transition and dissemination. Notably, silencing STING or ZEB1, or pharmacologically restoring autophagy, significantly suppressed tumor growth and metastasis. Conclusions: iMWA drives malignant progression of lung cancer through an mtDNA–cGAS–STING–ZEB1 signaling axis. Targeting this pathway—by inhibiting STING or enhancing autophagy—may represent a promising therapeutic strategy to mitigate recurrence and metastasis following microwave ablation.

## Simple Summary

Doctors often use heat treatments to destroy lung tumors, but if some cancer cells survive, the tumor frequently grows back faster and spreads aggressively. The reasons for this dangerous rebound are not fully understood. This study aims to uncover the exact biological chain of events that makes these surviving cells so harmful. The researchers discovered that incomplete heat damage causes DNA to leak inside the cells, triggering a specific internal “alarm” system that actively drives the cancer to spread. Identifying this hidden mechanism provides a crucial breakthrough for the medical community. It suggests that by pairing heat treatments with new drugs that block this alarm system or help clear the leaked DNA, doctors could prevent the cancer from returning, ultimately making these lung cancer therapies much safer and more effective for patients.

## 1. Introduction

Lung cancer is the leading cause of cancer-related deaths worldwide, with non-small-cell lung cancer (NSCLC) being the most prevalent pathological type [1,2]. For patients with early-stage lung cancer who are ineligible for surgery owing to comorbidities or poor functional status, local thermal ablation techniques, such as radiofrequency ablation, have become important alternative treatments [3,4]. Microwave ablation (MWA), a form of thermal ablation, uses high-frequency electromagnetic waves to induce frictional heat among water molecules within tissues, resulting in coagulative necrosis of tumor cells [5]. Although MWA exhibits promising potential in lung cancer treatment, its high local recurrence rate remains a serious challenge [6,7]. It must be emphasized that while complete ablation is always the clinical goal, incomplete microwave ablation (iMWA) frequently occurs as an unintended but unavoidable complication due to technical limitations, such as large tumor sizes, irregular margins, or the heat-sink effect from adjacent blood vessels. Numerous clinical reports suggest that residual tumors after incomplete microwave ablation (iMWA) may exhibit a more aggressive phenotype, accelerating disease progression [8,9,10,11]. Experimental research has further proven that sublethal thermal exposure might enhance the proliferation and metastasis of residual hepatocellular carcinoma cells [12,13,14]; however, the mechanisms behind lung cancer remain incompletely elucidated.

Recent research has revealed that various cancer therapies (radiotherapy and chemotherapy) may trigger tumor cell death and release damage-associated molecular patterns (DAMPs), such as mtDNA, thereby activating the cGAS-STING innate immune signaling pathway [15,16]. The cGAS-STING pathway plays a crucial role in both infection and tumor immunity. Under normal circumstances, its activation induces type I interferon expression and promotes anti-tumor immune responses [17,18]. However, the persistent activation of STING signaling inside tumor cells has been demonstrated to boost NF-κB-dependent pro-survival signals, thereby promoting tumor progression [15,19]. Notably, in KRAS-driven lung cancer, epigenetic silencing of STING signaling is a crucial mechanism of tumor immune evasion [15]. Conversely, radiotherapy research has revealed that radiation-induced DNA damage can result in cytosolic mtDNA accumulation, which in turn mediates radiation-induced lung injury through the cGAS-STING pathway [16], indicating a dual role of this pathway in lung cancer therapy.

This study elucidates the molecular mechanisms by which iMWA facilitates lung cancer progression, focusing on tumor cell-autonomous cGAS-STING signaling. Using in vivo and in vitro iMWA models to mimic residual tumor behavior, we examined the role of the mtDNA-cGAS-STING axis and its downstream effectors. Our findings provide a novel theoretical foundation for improving iMWA efficacy and suppressing recurrence.

This study complies with the TITAN 2025 Guidelines for transparency in the reporting and use of artificial intelligence [20].

## 2. Materials and Methods

### 2.1. Animal Model and iMWA Procedure

Four- to six-week-old male nude mice were used for in vivo studies. H1650 cells (5 × 10^6^) were subcutaneously injected into the right flank. When tumor volume reached approximately 150 mm^3^, mice were randomly assigned to the sham or iMWA groups (*n* = 5 per group) using a random number generator. Microwave ablation was performed under anesthesia using a clinical ECO-100A1 Microwave Ablation System (ECO Medical Instrument, Nanjing, China) at 1 W for 180 s [4,7,21]. The sham group underwent antenna insertion without energy delivery. Tumors and peripheral blood were collected 14 days post-ablation. All experiments were approved by the Institutional Animal Care and Use Committee.

### 2.2. Cell Culture and Heat Treatment

Two human NSCLC cell lines, H1650 (Cat. No.ZQ0385) and H1975 (Cat. No.ZQ0395), were purchased from Shanghai Zhongqiao Xinzhou Biotechnology Co., Ltd. (Shanghai, China) and were cultured in RPMI-1640 with 10% FBS. To simulate iMWA in vitro, cells underwent a 47 °C water bath for 5 min, recovered to 80% confluence, and were then treated for an additional 10 min. Cells that survived the treatment were designated as the HT group [7,21,22,23,24]. Control cells (NC) were maintained at 37 °C.

### 2.3. RNA Interference and Transfection

Lentiviral vectors encoding shRNAs targeting human STING and ZEB1 were designed. Stable knockdown cell lines were selected using puromycin (2 µg/mL). Scrambled shRNA was employed as a negative control.

### 2.4. RNA Extraction and Quantitative Real-Time PCR (qRT-PCR)

Total RNA was extracted using TRIzol reagent (Invitrogen, Carlsbad, CA, USA). cDNA was synthesized with PrimeScript RT Master Mix(Takara Bio Inc., Kusatsu, Japan). qRT-PCR was performed using SYBR Green on a QuantStudio system(Thermo Fisher Scientific, Waltham, MA, USA). GAPDH served as an internal control.

### 2.5. Western Blot

Proteins extracted with RIPA buffer were separated by SDS-PAGE and transferred to PVDF membranes. After blocking, membranes were incubated overnight at 4 °C with primary antibodies (cGAS, STING, ZEB1, LC3B, P62, PINK1, GAPDH), followed by HRP-conjugated secondary antibodies. Signals were visualized using ECL. Uncropped Western blot images are shown in File S1.

### 2.6. Immunofluorescence (IF) and Immunohistochemistry (IHC)

For IF, cells or frozen sections were fixed, permeabilized, and incubated with antibodies against STING, mtDNA, TOM20, or LC3B. Nuclei were stained with 4′,6-diamidino-2-phenylindole (DAPI). For IHC, paraffin sections were dewaxed, antigen-retrieved, and incubated with ZEB1 antibody. Images were captured using a confocal microscope.

### 2.7. Cell Proliferation, Migration, and Invasion Assays

Proliferation was measured by a CCK-8 assay and RTCA. Migration was evaluated using a scratch wound healing assay. Invasion was assessed by Transwell chambers pre-coated with Matrigel.

### 2.8. mtDNA Quantification and Manipulation

Intracellular and extracellular mtDNA levels were measured using the Quant-iT PicoGreen assay. For mtDNA depletion, cells were treated with EtBr (10 µg/mL) for 48 h. Exogenous mtDNA was introduced using Poly(dA:dT) at a concentration of 1 µg/mL.

### 2.9. Transcriptome Sequencing and Bioinformatic Analysis

DT and NC cells were subjected to RNA sequencing using the Illumina NovaSeq 6000 platform. Raw reads were subjected to quality control using FastQC and normalized via the TPM (transcripts per million) method. DEGs were identified with a threshold of |log_2_FC| > 1 and adjusted *p* < 0.05. GO enrichment analysis was performed.

### 2.10. Autophagy and Mitophagy Analysis

mt-Keima Assay: Cells were transduced with mt-Keima lentivirus (pH-sensitive fluorescent protein targeted to mitochondria) at an MOI of 20 for 48 h. After heat treatment, the cells were analyzed using a confocal microscope with dual-excitation wavelengths (458 nm for neutral pH, 561 nm for acidic pH). The ratio of 561/458 nm fluorescence was quantified to assess mitophagic flux.

JC-1 Staining: The cells were incubated with JC-1 dye (5 µg/mL) at 37 °C for 30 min. Mitochondrial membrane potential was evaluated by measuring the ratio of red (aggregates, ~590 nm) to green (monomers, ~529 nm) fluorescence using flow cytometry or fluorescence microscopy. A decrease in the red/green ratio indicates mitochondrial depolarization.

### 2.11. CTC Enumeration

Peripheral blood was collected and analyzed using the CellSearch^®^ system (Menarini Silicon Biosystems, Huntingdon Valley, PA, USA) according to the manufacturer’s protocol.

### 2.12. Statistical Analysis

All in vitro independent experiments were performed in biological triplicate (*n* = 3). Data are presented as mean ± standard deviation. Data normality was assessed using the Shapiro–Wilk test. Comparisons between two groups were analyzed using Student’s *t*-test. Multiple groups were compared using one-way analysis of variance followed by Tukey’s test. Statistical significance was set at * *p* * < 0.05.

### 2.13. Bioinformatic Analysis of Public Transcriptomic Data

Raw transcriptomic data from two independent datasets (GSE269585 and GSE279569) were downloaded from the GEO database. Differential expression analysis between HT and NC samples was performed using the limma package in R, with thresholds set at |log_2_FC| > 1 and adjusted * *p* * < 0.05. The DEGs between the two datasets were identified by taking the intersection of significantly dysregulated genes.

### 2.14. Functional Enrichment and GSVA Scoring

GO biological process and KEGG pathway enrichment analyses of the standard DEG set were conducted using the clusterProfiler package. GSVA was performed on in-house-generated transcriptomic data (DT versus NC) using the 80-gene signature set with the GSVA R package. Enrichment scores were compared between groups using the Wilcoxon test.

### 2.15. Functional Enrichment Chord Plot

Functional chord plots were generated to visualize the association between significantly enriched terms or pathways and their corresponding genes using the circlize package in R. Genes were ranked based on their enrichment significance and fold change.

### 2.16. mtDNA Isolation and Quantification

mtDNA was isolated from culture supernatants (via differential centrifugation) and cytosolic fractions (via 0.005% digitonin permeabilization). DNA was extracted using the DNeasy Blood & Tissue Kit (QIAGEN, Hilden, Germany) and enriched for mtDNA (Abcam, Cambridge, UK). Copy numbers were quantified via TaqMan qPCR targeting mt-ATP6, calculated using a plasmid standard curve, and normalized to nuclear β-actin.

All animal experiments were reported in accordance with the ARRIVE guidelines [25].

#### 2.16.1. Theory/Calculation

Incomplete microwave ablation (iMWA) establishes a distinct pathological niche defined by central necrosis and peripheral tumor cell survival. Sublethal thermal stress triggers necrotic cell death and the release of damage-associated molecular patterns, particularly double-stranded DNA (mtDNA), while simultaneously impairing autophagic and mitophagic clearance in surviving cells. The resulting cytosolic accumulation of mitochondrial and extracellular mtDNA engages the intrinsic cyclic GMP–AMP synthase (cGAS)–stimulator of interferon genes (STING) pathway.

In contrast to its canonical role in anti-tumor immunity, persistent activation of cGAS–STING within tumor cells elicits NF-κB- and EMT-related transcriptional programs. Among these, the transcription factor ZEB1 emerges as a critical downstream effector, driving epithelial–mesenchymal transition, invasive dissemination, and therapeutic resistance.

This theoretical framework integrates cellular stress, innate immune signaling, and phenotypic plasticity to explain the malignant evolution of residual tumors following iMWA. Transcriptomic enrichment of DNA-sensing and interferon-response pathways supports the activation of this axis. Clinically, these insights suggest that modulation of the cGAS–STING–ZEB1 circuit—or restoration of autophagic flux—may represent a rational strategy to suppress post-ablation recurrence and improve therapeutic durability in lung cancer.

#### 2.16.2. Highlights

Incomplete microwave ablation enhances lung cancer growth and metastasis.
Thermal injury triggers mtDNA release and activates the cGAS–STING pathway.
STING upregulates ZEB1 to drive epithelial–mesenchymal transition.
Autophagy inhibition amplifies cytosolic mtDNA and pathway activation.
Targeting the cGAS–STING–ZEB1 axis may prevent post-ablation recurrence.

## 3. Results

### 3.1. Incomplete Ablation Promotes Growth and Invasion of Lung Cancer

**Figure 1:**
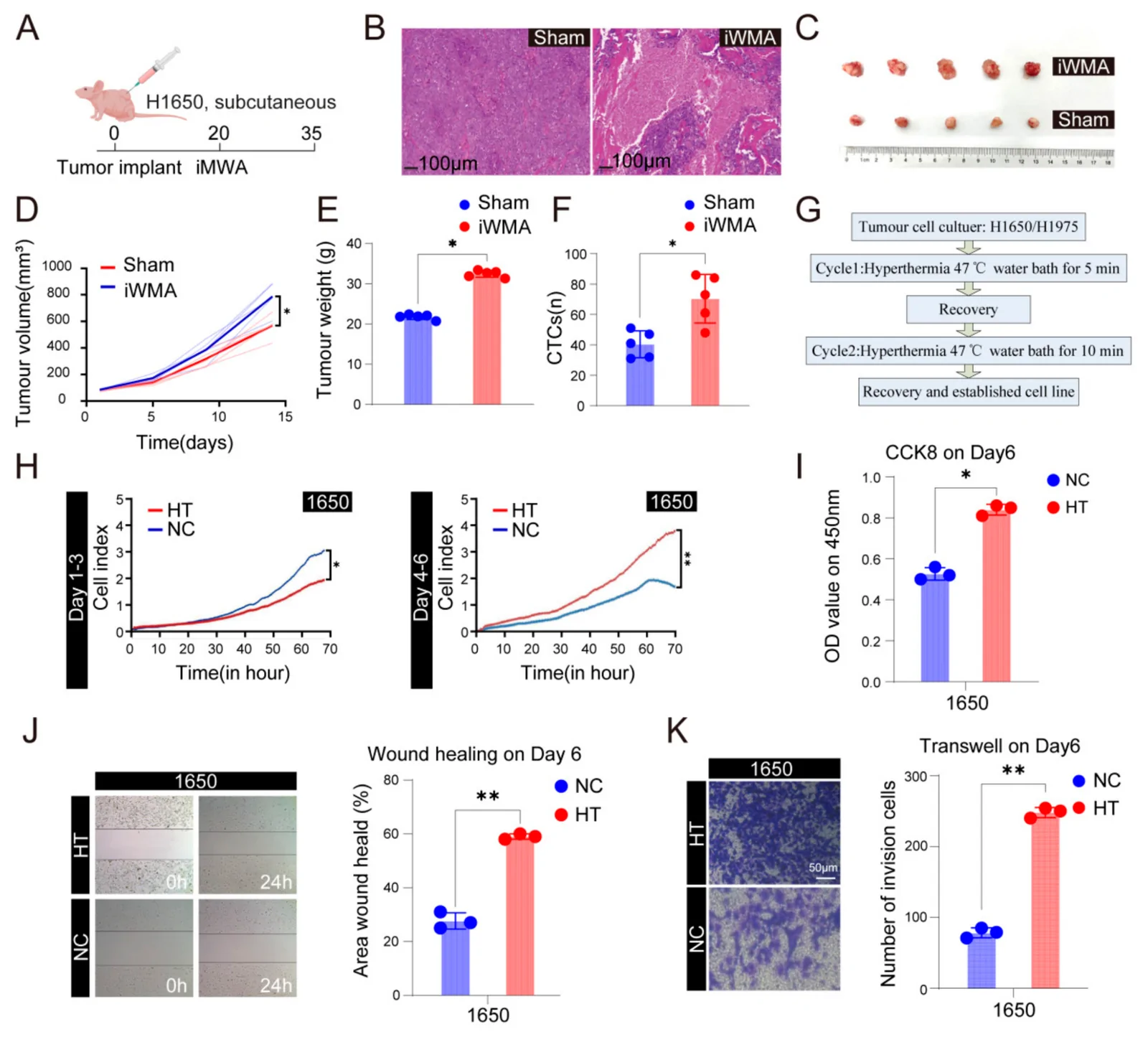
iMWA promotes aggressive behavior of lung cancer cells in vivo and in vitro. (**A**) Schematic illustration of the establishment of the in vivo iMWA model using H1650 cells. (**B**) Representative H&E-stained sections of tumors from sham and iMWA groups. Scale bar: 100 μm. (**C**) Representative photographic images of excised tumors from sham and iMWA groups on post-operative day 14. Scale bar: 1 cm. (**D**) Growth curves of tumors in sham and iMWA groups. Tumor volume was calculated as V = 0.5 × length × width2. Data are presented as mean ± SEM. (**E**) Weights of tumors harvested from sham and iMWA groups at the endpoint. (**F**) Counts of CTCs in the peripheral blood of mice in sham and iMWA groups. (**G**) Schematic diagram of the in vitro model simulating iMWA via water bath heating: the HT group underwent sequential heat treatments at 47 °C (5 min, 10 min) with a recovery interval, while the NC group was maintained at 37 °C. (**H**) Cell proliferation analysis of H1650 cells using RTCA. The cell index was monitored for days 1–3 (**left**) and days 4–6 (**right**) in the NC and HT groups. (**I**) Cell viability of H1650 cells measured by CCK-8 assay on day 6. (**J**) Wound healing assay in H1650 cells. Representative images (**left**) and quantitative analysis of the wound healed area (**right**) on day 6. (**K**) Transwell invasion assay in H1650 cells. Representative images (**left**) and quantitative analysis of the number of invaded cells (**right**) on day 6. Scale bar: 50 μm. * *p* < 0.05, ** *p* < 0.01. Abbreviations: iMWA, incomplete microwave ablation; Sham, sham-operated group; H&E, hematoxylin and eosin; CTC, circulating tumor cell; HT, heat-treated group; NC, normal control group; RTCA, real-time cell analysis; CCK-8, Cell Counting Kit-8.

To investigate the impact of iMWA on lung cancer progression, we established an in vivo iMWA model. H1650 cells were subcutaneously inoculated into nude mice, and microwave ablation (1 W, 180 s) was initiated when the tumor volume reached approximately 150 mm^3^ [4,7,21]. The sham-operated group (sham) underwent an identical procedure, including antenna insertion, however, without energy delivery. Samples were collected 14 days post-operation (Figure 1A). Hematoxylin and eosin (H&E) staining revealed a heterogeneous structure in the iMWA-group tumors, characterized by central necrosis and a peripheral zone of residual viable tumor cells (Figure 1B), in contrast to the homogeneous structure observed in the sham group, confirming the successful establishment of the model. Notably, tumor volume (Figure 1C,D) and weight (Figure 1E) were significantly higher in the iMWA group than in the sham group, and the number of circulating tumor cells (CTCs) in peripheral blood (Figure 1F) was markedly increased.

The thermal effect of iMWA was stimulated in vitro by subjecting cells to sequential heat treatments in a water bath at 47 °C, first for 5 min, and then 10 min, after recovery to analyze the malignant biological behavior of residual tumor cells (Figure 1G) [7,22,23,24]. The scratch assay indicated that the migration ability of the heat-treated (HT) group was significantly enhanced on day 6 (Figure 1J, left; Supplementary Figure S1C), and the wound healing rate was significantly higher than that of the normal control (NC) group (Figure 1J, right; Supplementary Figure S1D). Furthermore, real-time cell analysis (RTCA) curves indicated that proliferation in the HT group was initially inhibited within the first 72 h; however, it surpassed that of the NC group from days 4 to 6 (Figure 1H; Supplementary Figure S1A). The CCK-8 assay further confirmed a significant increase in cell viability in the HT group on day 6 (Figure 1I; Supplementary Figure S1B). The Transwell assay consistently demonstrated an enhanced invasive ability in the HT group (Figure 1K; Supplementary Figure S1E). These findings suggest that residual tumor cells after iMWA exhibit enhanced migratory, proliferative, and invasive capacities, potentially driving local recurrence and systemic metastasis.

### 3.2. Incomplete Ablation Promotes Lung Cancer Progression by Activating the cGAS-STING Signaling Pathway

**Figure 2:**
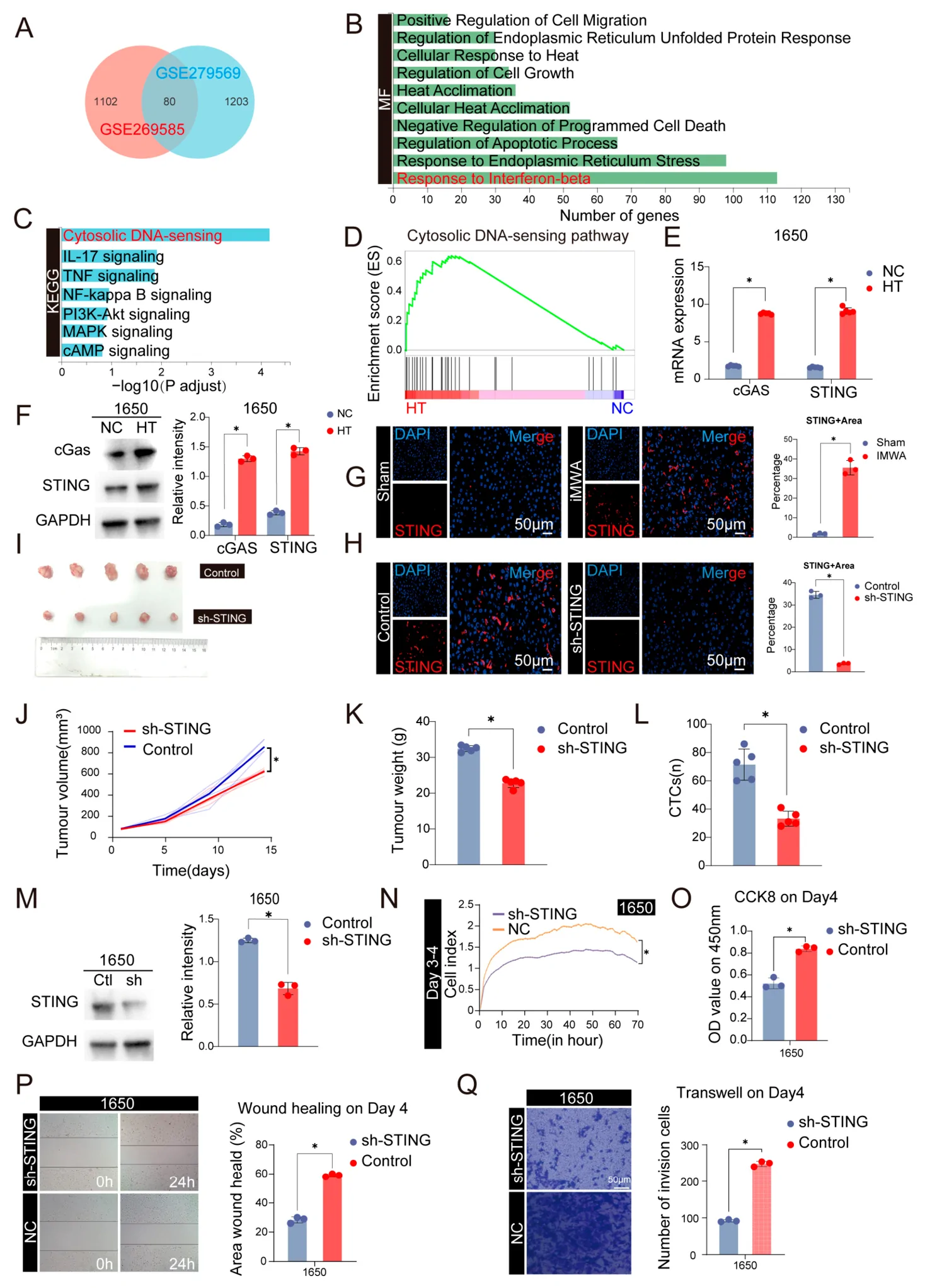
STING activation in residual tumor cells mediates the pro-tumorigenic effects of iMWA. (**A**) Identification of common DEGs (80 genes) from two independent GEO datasets (GSE269585 and GSE279569, HT versus NC) by intersection analysis. (**B**) GO enrichment analysis of the common gene set, showing the top enriched pathways including “Positive Regulation of Cell Migration”. (**C**) KEGG pathway enrichment analysis indicating the activation of the “Cytosolic DNA-sensing pathway”. (**D**) GSEA enrichment plot confirming upregulation of the cytosolic DNA-sensing pathway in the HT group. (**E**) qRT-PCR analysis of cGAS and STING mRNA expression in H1650 cells after heat treatment (HT) compared with NC cells. (**F**) Western blot analysis of cGAS and STING protein levels (**left**) and quantitative analysis (**right**) in NC and HT H1650 cells. GAPDH serves as the loading control. (**G**) IF staining for STING (red) in tumor tissues from the sham and iMWA groups. Nuclei are counterstained with DAPI (blue). Scale bar: 50 μm. The right panel shows the quantification of STING+ area. (**H**) IF staining validating STING knockdown in control and sh-STING H1650 cells (red). Scale bar: 50 μm. The right panel shows the quantification of STING+ area. (**I**) Representative photographic images of excised tumors from mice xenografted with control or sh-STING cells. (**J**) Tumor growth curves of xenografts from control and sh-STING groups over time. (**K**) Final weights of tumors harvested from control and sh-STING groups at the endpoint. (**L**) Counts of CTCs in the peripheral blood of mice bearing control or sh-STING xenografts. (**M**) Western blot analysis of STING knockdown efficiency (**left**) and quantitative analysis (**right**) in H1650 cells. (**N**) Proliferation analysis using RTCA in NC and sh-STING H1650 cells, highlighting the divergence in cell index during days 3–4. (**O**) Cell viability measured by CCK-8 assay on day 4 in control and sh-STING H1650 cells. (**P**) Wound healing assay performed on day 4 in H1650 cells. Representative images at 0 h and 24 h (**left**) and quantitative analysis of the wound healed area (**right**). (**Q**) Transwell invasion assay performed on day 4 in H1650 cells. Representative images (**left**) and quantitative analysis of invaded cells (**right**). * *p* < 0.05 Abbreviations: DEG, differentially expressed gene; GEO, Gene Expression Omnibus; HT, heat-treated; NC, normal control; GO, Gene Ontology; KEGG, Kyoto Encyclopedia of Genes and Genomes; GSEA, gene set enrichment analysis; qRT-PCR, quantitative real-time polymerase chain reaction; cGAS, cyclic GMP-AMP synthase; STING, stimulator of interferon genes; IF, immunofluorescence; iMWA, incomplete microwave ablation; DAPI, 4′,6-diamidino-2-phenylindole; sh, short hairpin RNA; CTC, circulating tumor cell.

To investigate the molecular regulatory mechanisms of iMWA, we initially conducted a systematic bioinformatic analysis of publicly available transcriptomic data from microwave-based heat treatment. Two independent lung cancer cell datasets (GSE269585 and GSE279569) were acquired from the Gene Expression Omnibus (GEO) database. A differential expression analysis between HT and NC groups was conducted, yielding 80 common differentially expressed genes (DEGs) (Figure 2A). Furthermore, GO enrichment analysis of these DEGs highlighted significant alterations in cellular components and biological processes related to extracellular matrix organization and ubiquitin-related functions (Supplementary Figure S2A). Functional enrichment analysis of this gene set revealed a significant enrichment in the “Response to Interferon-beta” pathway based on Gene Ontology (GO) biological process analysis (Figure 2B). Additionally, Kyoto Encyclopedia of Genes and Genomes (KEGG) pathway analysis indicated a marked activation of the “Cytosolic DNA-sensing pathway” (Figure 2C). Gene set enrichment analysis (GSEA) further confirmed upregulation of the cytosolic DNA-sensing pathway (Figure 2D). These bioinformatic results suggest that heat treatment may activate interferon-related pathways through the cytosolic DNA-sensing mechanism.

Based on these findings, we hypothesized that microwave ablation induces tumor cell necrosis through thermal effects, potentially activating the cGAS-STING pathway by releasing intracellular mtDNA from necrotic cells. Previous studies have found that various treatments, including radiotherapy, chemotherapy, and DDR inhibitors, can activate anti-tumor immunity through this pathway [17,26]. Our study hypothesized that DAMPs, particularly mtDNA released from necrotic cells, may be efficiently internalized by adjacent surviving tumor cells, given the distinctive spatial structure of “central necrosis and peripheral survival” that forms after microwave ablation therapy. Along with reports that autonomous STING signaling in tumor cells drives malignant progression [27,28], we proposed that internalized necrotic mtDNA in residual tumor cells after iMWA may promote a malignant phenotype through tumor-intrinsic STING signaling.

To test this hypothesis, we examined changes in the cGAS–STING pathway in heat-treated lung cancer cells. qRT-PCR analysis demonstrated significant upregulation of cGAS and STING mRNA expression in heat-treated H1650 cells (Figure 2E), with consistent results observed in H1975 cells (Supplementary Figure S2B). Western blot analysis further confirmed increased cGAS and STING protein levels in H1650 cells following heat treatment (Figure 2F), which was similarly validated in H1975 cells (Supplementary Figure S2C). In vivo immunofluorescence staining revealed elevated STING expression in the marginal regions of tumors from the iMWA group (Figure 2G).

STING knockdown using shRNA was validated by immunofluorescence and Western blot analysis in H1650 cells (Figure 2H,M) and in H1975 cells (Supplementary Figure S2D). Functional in vivo assays showed that STING knockdown significantly suppressed tumor growth (Figure 2I–K) and reduced circulating tumor cell (CTC) counts (Figure 2L). In vitro, STING knockdown markedly attenuated cell proliferation in H1650 cells, as evidenced by RTCA and CCK-8 assays (Figure 2N,O), with comparable inhibitory effects observed in H1975 cells (Supplementary Figure S2E). Moreover, STING depletion significantly impaired migratory and invasive capacities of H1650 cells (Figure 2P,Q), which was consistently reproduced in H1975 cells (Supplementary Figure S2F,G). These findings demonstrate that mtDNA released from necrotic tumor cells induces malignant phenotypes by activating the cGAS–STING pathway in residual tumor cells.

### 3.3. Incomplete Ablation Promotes mtDNA Release from Lung Cancer Cells to Activate the cGAS–STING Signal

**Figure 3:**
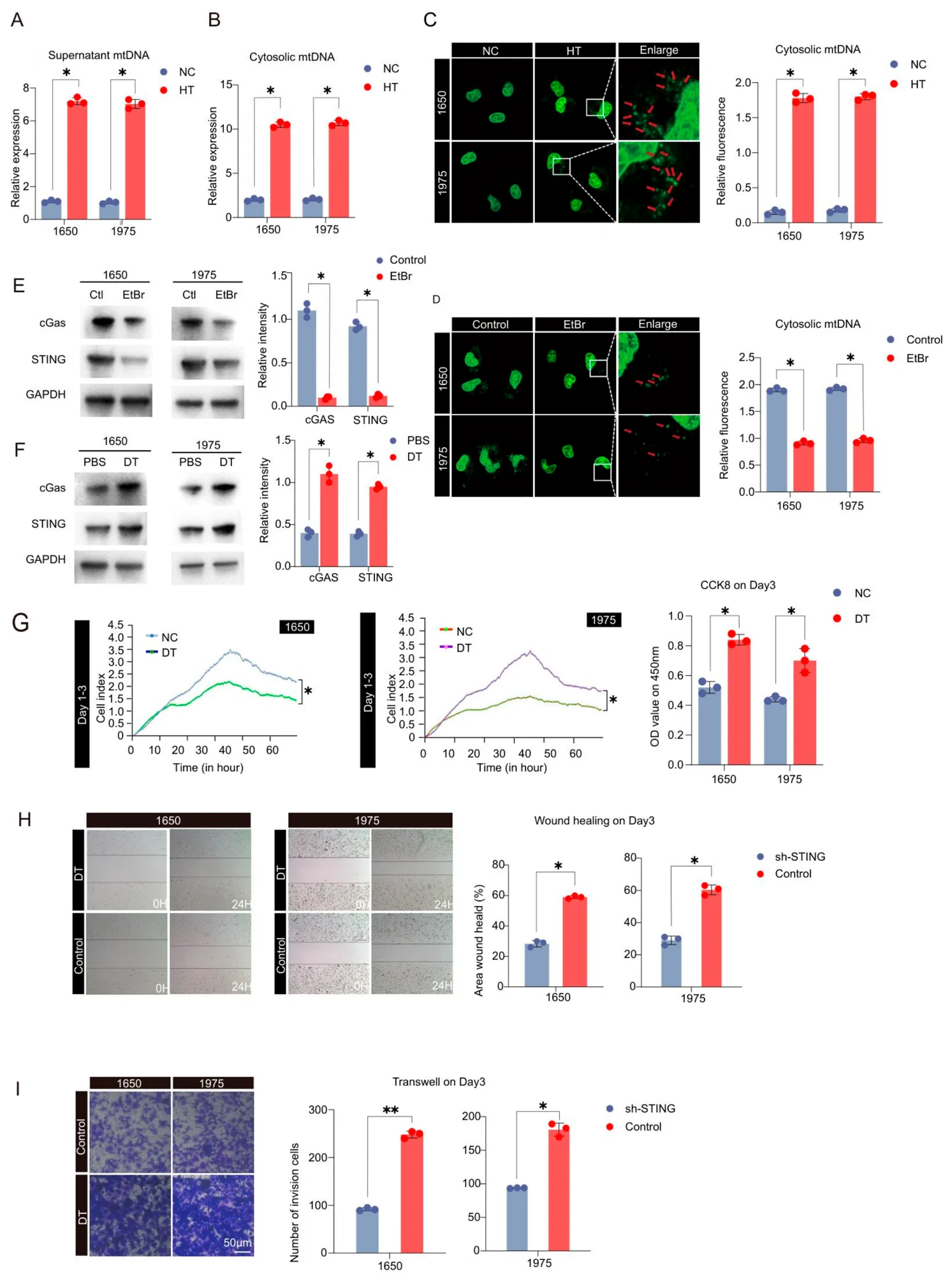
Necrotic mtDNA released after thermal injury activates the cGAS-STING pathway to promote malignant phenotypes. (**A**) Quantification of free mtDNA levels in the cell culture supernatants of H1650 and H1975 cells (NC and HT groups), measured using the PicoGreen assay. (**B**) Quantification of cytosolic free mtDNA levels in H1650 and H1975 cells (NC and HT groups) using the PicoGreen assay. (**C**) IF detection of intracellular mtDNA (green) in H1650 and H1975 cells (NC and HT groups). Red arrows indicate cytosolic mtDNA puncta in the enlarged images. The right panel shows the quantification of fluorescence intensity. (**D**) IF analysis of intracellular mtDNA (green) in control and EtBr-treated H1650 and H1975 cells. Red arrows indicate cytosolic mtDNA. The right panel shows the quantification of fluorescence intensity. (**E**) Western blot analysis of cGAS and STING protein expression (**left**) and quantitative analysis (**right**) in control and EtBr-treated H1650 cells. (**F**) Western blot analysis of cGAS and STING protein expression (**left**) and quantitative analysis (**right**) in PBS control and DNase I-treated (DT) H1650 cells. (**G**) Cell proliferation analysis: RTCA curves monitoring days 1–3 (left/middle) and CCK-8 assay results on day 3 (right) for NC and DT groups in H1650 and H1975 cells. (**H**) Wound healing assay performed on day 3. Representative images at 0 h and 24 h (**left**) and quantitative analysis of the wound healed area (**right**) for NC and DT groups in H1650 and H1975 cells. (**I**) Transwell invasion assay performed on day 3. Representative images of crystal violet-stained invaded cells (**left**) and quantitative analysis of invaded cells (**right**) for NC and DT groups in H1650 and H1975 cells. Scale bar: 50 μm. * *p* < 0.05, ** *p* < 0.01.Abbreviations: mtDNA, double-stranded DNA; NC, normal control; HT, heat-treated; IF, immunofluorescence; DAPI, 4′,6-diamidino-2-phenylindole; EtBr, ethidium bromide; PBS, phosphate-buffered saline; DT, DNase I-treated group; RTCA, real-time cell analysis; CCK-8, Cell Counting Kit-8.

The current study observed that the levels of free mtDNA were significantly elevated in both the cell culture supernatants and cytosol after heat treatment in H1650 and H1975 cells (Figure 3A,B), suggesting that thermal injury induces mtDNA release and cytosolic accumulation within residual surviving cells. Immunofluorescence staining further revealed a substantial accumulation of mtDNA in the cytoplasm following heat treatment (Figure 3C). Intracellular mtDNA was depleted using ethidium bromide (EtBr), which markedly reduced cytosolic mtDNA signals, confirming the essential role of intracellular mtDNA in activating the cGAS–STING pathway (Figure 3D). Given that cytosolic mtDNA leakage is closely associated with activation of the cGAS–STING pathway, we next examined the effect of EtBr treatment on cGAS and STING expression. Consistently, EtBr treatment significantly decreased the protein levels of cGAS and STING (Figure 3E).

To further evaluate the contribution of extracellular mtDNA to cGAS–STING activation, extracellular DNA was degraded by DNase I treatment (DT). DNase I-mediated depletion of extracellular mtDNA significantly attenuated cGAS and STING protein expression (Figure 3F), accompanied by suppressed proliferative capacity, as determined by RTCA and CCK-8 assays (Figure 3G). In parallel, DNase I treatment markedly reduced the migratory and invasive abilities of tumor cells, as demonstrated by wound healing and Transwell invasion assays (Figure 3H,I).

Collectively, these results indicate that mtDNA released from necrotic tumor cells following thermal injury can be internalized by residual tumor cells, leading to activation of the cGAS–STING pathway and promotion of malignant phenotypes in the context of incomplete ablation.

### 3.4. Incomplete Ablation Promotes Lung Cancer Progression via the STING-ZEB1 Axis

**Figure 4:**
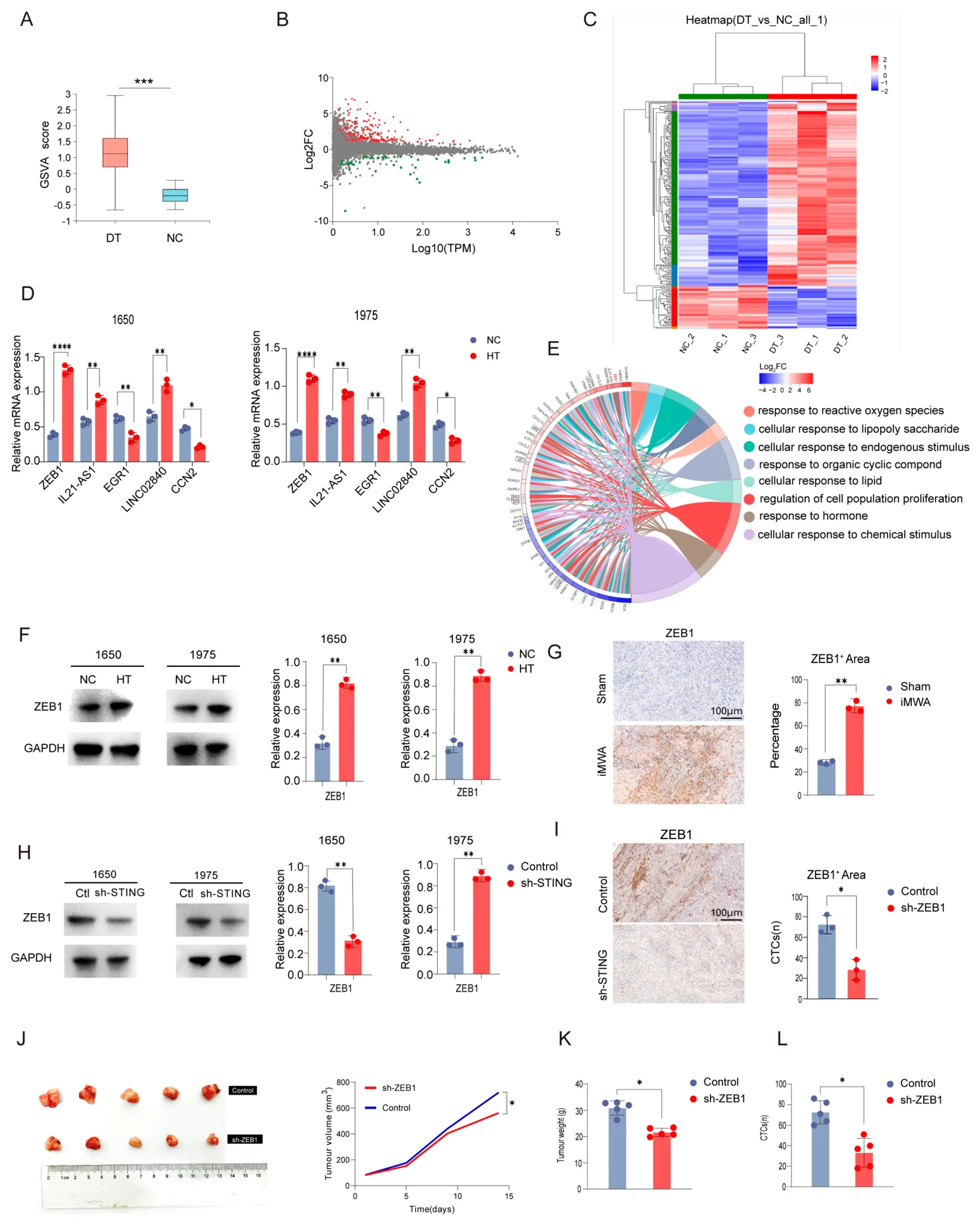
The cGAS-STING-ZEB1 axis promotes residual tumor progression after incomplete ablation. (**A**) GSVA score analysis of the “Response to Interferon-beta” pathway in DT (Poly(dA:dT)-treated) versus NC groups. (**B**) MA plot presenting DEGs between DT and NC groups from RNA-seq analysis. (**C**) Heatmap of significantly altered genes in DT compared to NC groups. (**D**) qRT-PCR validation of ZEB1 and other top upregulated genes in H1650 and H1975 cells (NC vs HT groups). (**E**) Functional enrichment chord plot of differential genes, highlighting ZEB1 and proliferation-related terms. (**F**) Western blot analysis of ZEB1 protein expression (**left**) and quantitative analysis (**right**) in NC and HT groups for H1650 and H1975 cells. (**G**) Representative IHC staining images of ZEB1 in tumor tissues from sham and iMWA groups (**left**) and quantitative analysis of ZEB1-positive area (**right**). Scale bar: 100 μm. (**H**) Western blot analysis of ZEB1 expression (**left**) and quantitative analysis (**right**) in control and sh-STING cells (H1650 and H1975). (**I**) Representative IHC staining images of ZEB1 in tumor tissues from control and sh-ZEB1 xenografts (**left**) and quantitative analysis of ZEB1-positive area (**right**). Scale bar: 100 μm. (**J**) Representative photographic images of excised tumors (**left**) and tumor growth curves (**right**) of control and sh-ZEB1 xenografts over time. (**K**) Final tumor weights in control and sh-ZEB1 groups at the experimental endpoint. (**L**) CTC counts in the peripheral blood of mice bearing control or sh-ZEB1 xenografts. * *p* < 0.05, ** *p* < 0.01, *** *p* < 0.001, **** *p* < 0.0001.Abbreviations: NC, normal control; DT, Poly(dA:dT)-treated group; HT, heat-treated group; iMWA, incomplete microwave ablation; DEGs, differentially expressed genes; GSVA, Gene Set Variation Analysis; IHC, immunohistochemistry; sh, short hairpin RNA; CTC, circulating tumor cell; TPM, transcripts per million.

To investigate the downstream mechanisms activated by cGAS–STING signaling following incomplete ablation, we performed Gene Set Variation Analysis (GSVA) on transcriptomic data derived from DT (Poly(dA:dT)-treated) and NC groups. GSVA revealed a significant enrichment of the “Response to Interferon-beta” pathway in the DT group compared with the NC group (Figure 4A), indicating effective activation of cGAS–STING-dependent inflammatory signaling.

We next analyzed differentially expressed genes (DEGs) between DT and NC groups using RNA sequencing. The MA plot demonstrated extensive transcriptional alterations induced by DT treatment (Figure 4B), and the heatmap further illustrated distinct gene expression patterns between the two groups (Figure 4C). Among the significantly upregulated genes, ZEB1 emerged as a prominent candidate. qRT-PCR validation confirmed that ZEB1 mRNA expression was markedly increased in both H1650 and H1975 cells following heat treatment compared with NC controls (Figure 4D). Functional enrichment chord plot analysis further highlighted ZEB1 as a highly ranked gene associated with proliferation- and invasion-related pathways (Figure 4E), suggesting its potential role as a key downstream effector.

Consistent with the transcriptional changes, Western blot analysis showed that ZEB1 protein levels were significantly elevated in the HT group compared with NC controls in both H1650 and H1975 cells (Figure 4F). In addition, immunohistochemical staining of tumor tissues from the in vivo iMWA model revealed markedly increased ZEB1 expression in the iMWA group relative to the sham group (Figure 4G). These results indicate that ZEB1 is a common downstream molecule activated following incomplete ablation both in vitro and in vivo.

To determine whether ZEB1 upregulation is dependent on cGAS–STING signaling, STING expression was silenced using shRNA. Western blot analysis demonstrated that knockdown of STING significantly reduced ZEB1 protein levels in both H1650 and H1975 cells (Figure 4H), supporting the notion that ZEB1 is regulated downstream of STING activation.

Finally, we assessed the functional significance of ZEB1 in residual tumor progression using a xenograft model. Compared with control tumors, sh-ZEB1 xenografts exhibited markedly reduced ZEB1 expression as confirmed by immunohistochemistry (Figure 4I), along with significantly suppressed tumor growth (Figure 4J), decreased final tumor weight (Figure 4K), and reduced circulating tumor cell (CTC) counts in peripheral blood (Figure 4L).

Collectively, these findings demonstrate that mtDNA-mediated activation of the cGAS–STING pathway after incomplete ablation promotes lung cancer growth and dissemination through induction of the ZEB1 axis.

### 3.5. Heat Treatment Regulates mtDNA Levels and Activates the cGAS-STING Pathway by Inhibiting Autophagy

Although the above results demonstrated that heat-induced necrotic cell death leads to mtDNA leakage, it remained unclear whether residual surviving tumor cells themselves also contribute to the increased mtDNA burden after heat treatment. Therefore, we next investigated alterations in autophagy and mitophagy pathways in tumor cells following thermal stress.

**Figure 5:**
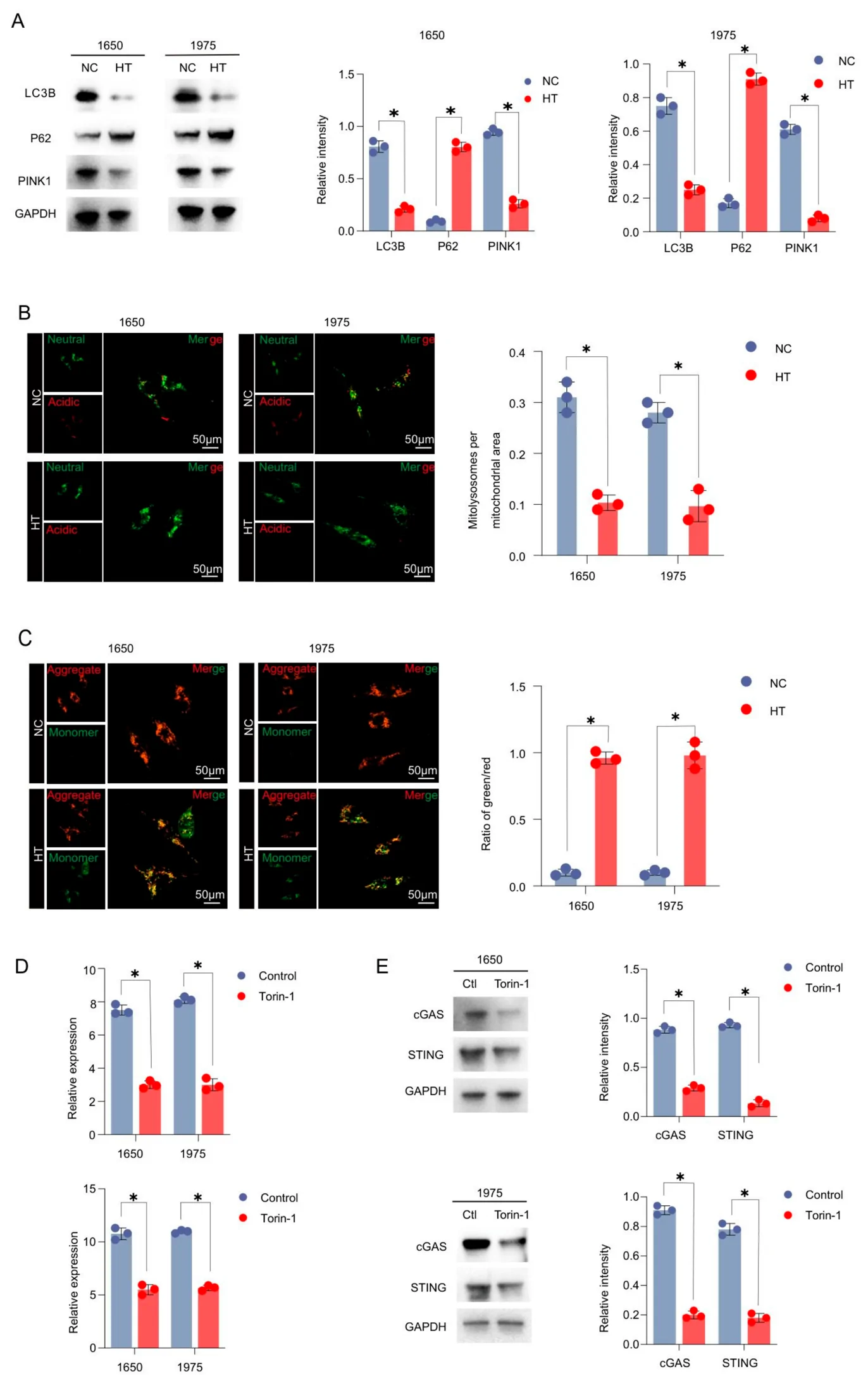
Heat treatment activates the cGAS-STING pathway by suppressing mitophagy and promoting mtDNA release. (**A**) Western blot analysis of autophagy-related proteins (LC3B, P62, and PINK1) in H1650 and H1975 cells (**left**) and quantitative analysis of relative protein intensity (**right**). GAPDH serves as a loading control. (**B**) Assessment of mitophagic flux using the mt-Keima reporter in H1650 and H1975 cells. Representative images show neutral environment (green, cytoplasm) and acidic environment (red, lysosomes) (**left**). Scale bar: 50 μm. The right panel shows the quantitative analysis of mitolysosomes per mitochondrial area. (**C**) Analysis of mitochondrial membrane potential using JC-1 staining. Representative images of H1650 and H1975 cells in NC and HT groups (**left**); red fluorescence indicates aggregates (high potential), and green fluorescence indicates monomers (low potential). Scale bar: 50 μm. The right panel illustrates the quantitative ratio of green/red fluorescence. (**D**) Measurement of mtDNA levels in the cell culture supernatant (**top**) and cytoplasm (**bottom**) of HT-treated H1650 and H1975 cells with or without Torin-1 treatment, using the PicoGreen assay. (**E**) Western blot analysis of cGAS and STING protein expression (**left**) and quantitative analysis (**right**) in HT-treated H1650 and H1975 cells with or without Torin-1. GAPDH serves as a loading control. Data is presented as mean ± SD. * *p* < 0.05. Abbreviations: NC, normal control; HT, heat treatment; LC3B, microtubule-associated protein 1 light chain 3 beta; PINK1, PTEN-induced kinase 1; cGAS, cyclic GMP-AMP synthase; STING, stimulator of interferon genes; mtDNA, double-stranded DNA; SD, standard deviation.

Western blot analysis revealed that heat treatment markedly altered the expression of autophagy-related proteins in both H1650 and H1975 cells, characterized by a decreased LC3B-II/I ratio and increased accumulation of P62 and PINK1 (Figure 5A), indicating impaired autophagic flux. To specifically assess mitophagy activity, we employed the mt-Keima reporter system. Heat-treated cells exhibited a significant reduction in mitolysosome formation compared with NC cells, as shown by decreased red fluorescence and quantitative analysis of mitophagic flux (Figure 5B). Consistently, JC-1 staining demonstrated a loss of mitochondrial membrane potential in heat-treated H1650 and H1975 cells, reflected by an increased green/red fluorescence ratio (Figure 5C), suggesting the accumulation of dysfunctional mitochondria.

Given that defective mitochondria are a major source of mitochondrial DNA (mtDNA), we next examined whether impaired mitophagy contributes to mtDNA accumulation. Activation of autophagy using the mTOR inhibitor Torin-1 prior to heat treatment significantly reduced mtDNA levels in both the cytoplasm and the cell culture supernatant of H1650 and H1975 cells (Figure 5D). Importantly, restoration of autophagic activity by Torin-1 also markedly attenuated the heat-induced upregulation of cGAS and STING proteins (Figure 5E), indicating that mitophagy inhibition is a key upstream event driving cGAS–STING activation following heat treatment.

Collectively, these findings reveal that heat treatment not only induces mtDNA release from necrotic cells but also suppresses mitophagy in residual surviving tumor cells, leading to abnormal accumulation and cytosolic leakage of mitochondrial mtDNA. This dual mechanism amplifies cGAS–STING signaling and thereby contributes to malignant progression after incomplete ablation.

## 4. Discussion

This study confirms that iMWA accelerates malignant progression in residual lung cancer via the “mtDNA-cGAS-STING-ZEB1” axis. Using in vitro and in vivo models, we demonstrated that the heat-damaged microenvironment triggers massive mtDNA release and cytoplasmic accumulation. This activates tumor cell-autonomous cGAS-STING signaling, enhancing invasion and metastasis. These findings identify novel therapeutic targets to prevent post-ablation recurrence. Unlike previous research that primarily concentrated on the role of this pathway in immune cells that induce type I interferon-mediated anti-tumor responses [29], our findings emphasize that the sustained activation of tumor cell-autonomous cGAS-STING signaling directly promotes tumor progression in the specific tumor microenvironment after iMWA, which is consistent with recent studies suggesting that “tumor cell-autonomous STING signaling promotes malignant progression.” This study provides the first comprehensive evidence chain linking thermal ablation to STING activation via mtDNA. We confirmed the necessity of mtDNA (using EtBr and Poly(dA:dT)) and validated STING’s functional role via knockdown. Notably, iMWA’s “central necrosis–peripheral survival” structure facilitates necrotic mtDNA internalization by surviving cells. This efficient activation of cell-autonomous STING signaling may represent a distinctive cancer-promoting mechanism unique to iMWA.

Additionally, this investigation identified ZEB1 as a critical downstream effector molecule of STING that mediates its role in promoting invasion and metastasis. Transcriptomic sequencing and subsequent validation experiments demonstrated that STING activation significantly upregulates ZEB1 expression, whereas STING inhibition reduces ZEB1 protein levels. The upregulation of ZEB1 expression directly explains the phenotypes of enhanced cell migration, invasion, and increased CTCs, since it is a major transcription factor in EMT. Importantly, the in vivo knocking down of ZEB1 significantly reduced the tumor development and metastasis induced by iMWA, hence confirming the necessity of ZEB1 in this malignant process [30].

Limitations of this study include the absence of human clinical specimens for validation due to ethical constraints. Additionally, the precise molecular mechanism linking cGAS–STING signaling to ZEB1 activation requires further elucidation.

The role of STING in tumor cells is receiving increasing attention, particularly in its contribution to tumor malignant progression. STING not only participates in the anti-tumor response through its impact on immune cells but also activates signaling pathways directly inside tumor cells to promote tumor cell survival, migration, and invasion. Research has demonstrated that the continuous activation of STING can collaborate with EMT-related transcription factors such as ZEB1 to facilitate tumor metastasis [31]. Subsequent research has also indicated that STING activation enhances tumor cell resistance to immune checkpoint inhibitors and other therapies by downregulating anti-tumor immune responses [17,32]. These findings are consistent with our results, which demonstrate that the sustained activation of STING in tumor cells not only enhances their immune evasion ability but also strengthens their migratory and invasive capabilities by promoting the EMT process.

Moreover, STING activation can promote immune escape in the tumor microenvironment by increasing the expression of other immunosuppressive factors. For example, cytokines such as IL-6 and TNF-α released upon STING pathway activation can induce polarization of tumor-associated macrophages into an immune-suppressive phenotype, further enhancing immune evasion and resistance to treatment [33]. This phenomenon is particularly evident in NSCLC, where abnormal STING pathway activation is closely linked to increased tumor immune escape.

Given the dual function of STING in tumor progression, targeting the STING–ZEB1 axis offers a potential therapeutic breakthrough. In clinical terms, for patients undergoing MWA with a high risk of residual margins, the prophylactic or adjuvant co-administration of STING inhibitors could block the acquired invasive traits of surviving cells. This combination of physical thermal ablation and targeted pharmacological inhibition could mitigate malignant progression during post-iMWA recurrence. Future studies should explore STING modulation to reshape the tumor microenvironment, leveraging inhibitors that have already shown promise in other malignancies to improve lung cancer outcomes.

In conclusion, this study systematically reveals a novel mechanism by which iMWA promotes the progression of residual lung cancer through inducing mtDNA release and autophagy inhibition, thereby activating the cGAS-STING-ZEB1 axis. This study not only provides a novel theoretical perspective for comprehending tumor recurrence after iMWA, but also suggests that intervention strategies targeting critical nodes of this pathway (STING or ZEB1) may offer novel treatment directions to prevent recurrence following ablation.

## 5. Conclusions

This study reveals a novel mechanism by which iMWA promotes lung cancer progression: insufficient thermal ablation causes mtDNA accumulation, which triggers a pro-tumorigenic signaling axis driven by the cGAS-STING-ZEB1 pathway in residual tumor cells. Targeting this pathway (for instance, via STING inhibition) may represent a potential therapeutic strategy to suppress tumor recurrence after microwave ablation.

## Data Availability

The research data can be made available upon reasonable request from the corresponding author.

## Supplementary Materials

The following supporting information can be downloaded at https://www.mdpi.com/article/10.3390/cancers18162594/s1. Figure S1: iMWA promotes aggressive behavior of H1975 lung cancer cells in vitro. Figure S2: STING activation in H1975 residual tumor cells mediates the pro-tumorigenic effects of iMWA. File S1: Original images.
