# Supplementary material for: Residual Lung Cancer After Incomplete Microwave Ablation Exhibits cGAS–STING–ZEB1-Driven Malignant Progression
Source: Cancers (Basel). 2026 Aug 12;18(16):2594. doi: 10.3390/cancers18162594 (PMC13511148; doi:10.3390/cancers18162594)
Supplement: Supplementary file 1 [file cancers-18-02594-s001.zip › File S1.pdf]

2F CGAS

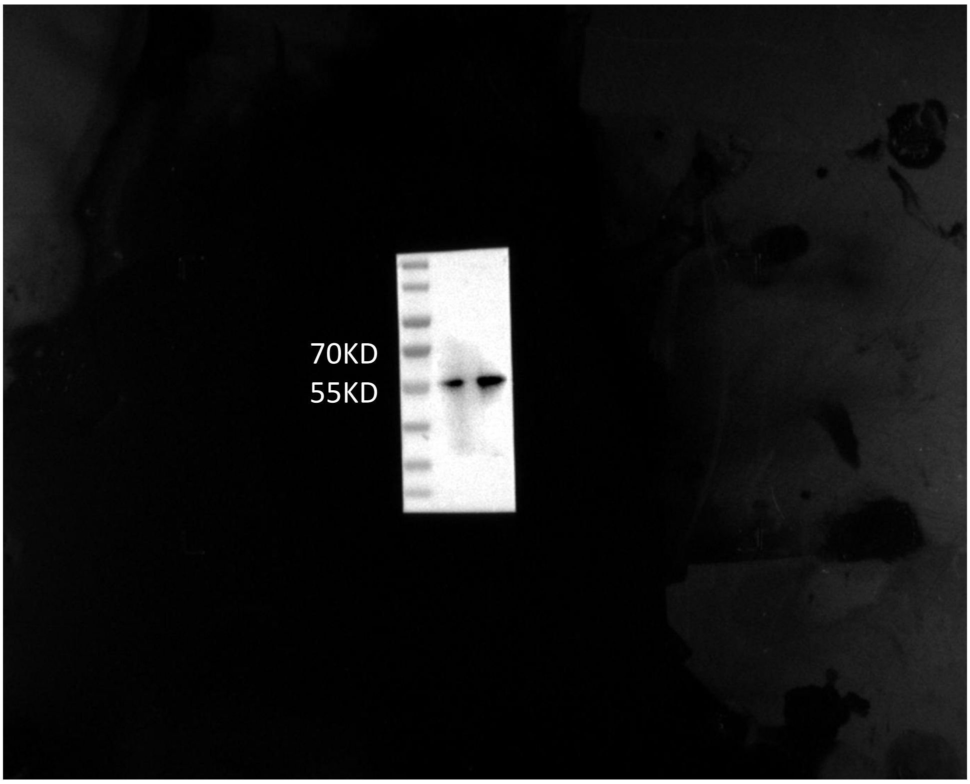

2F STING

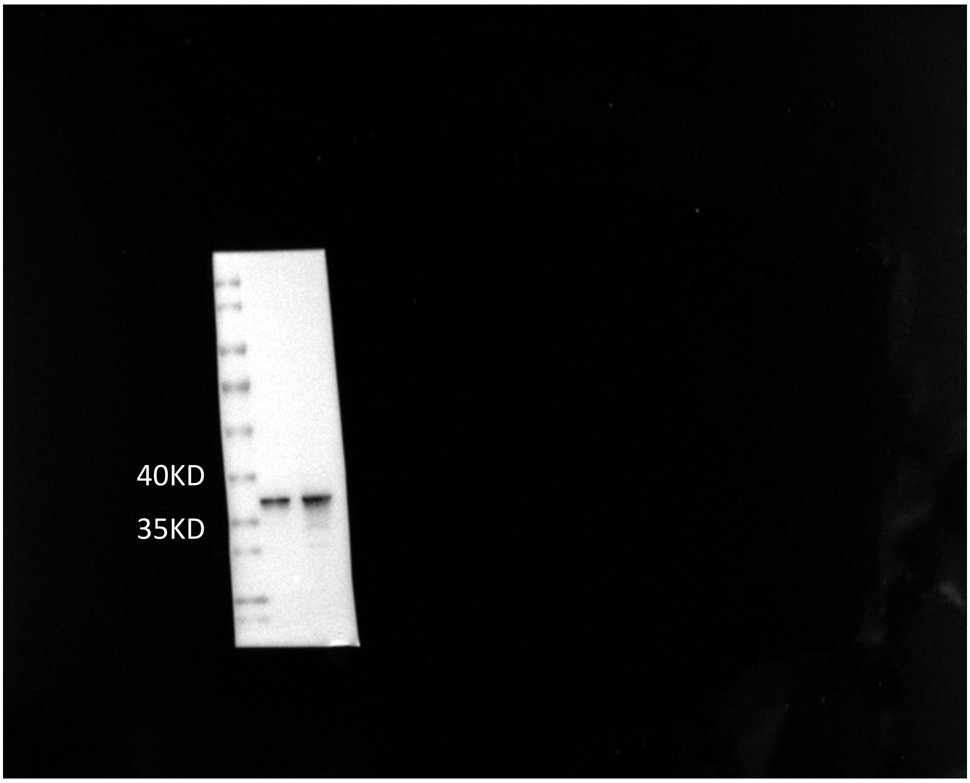

2F GAPDH

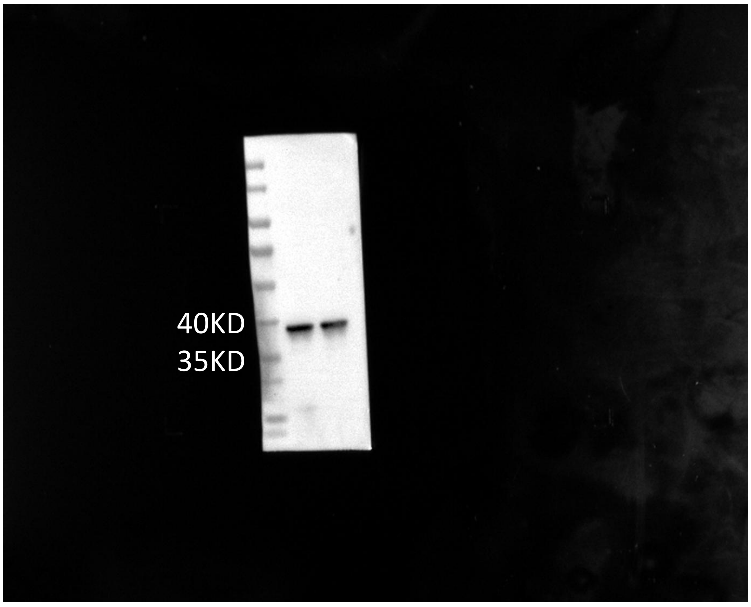

2M STING

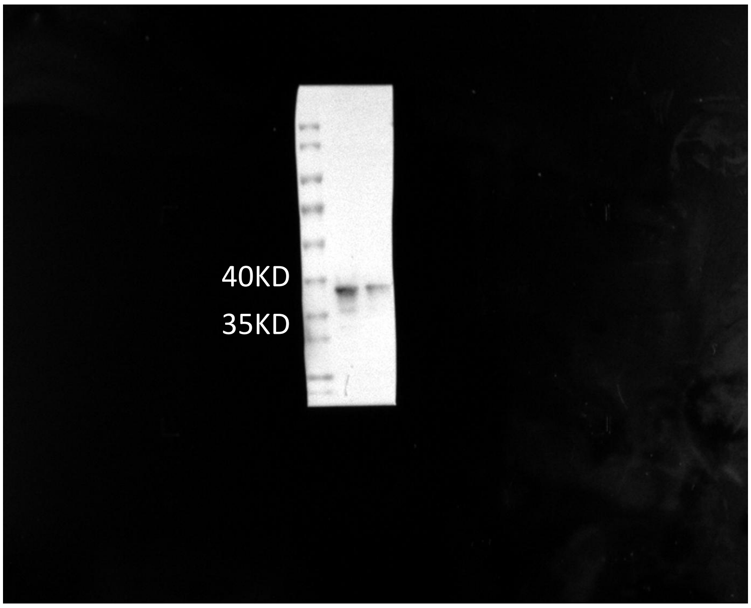

2M GAPDH

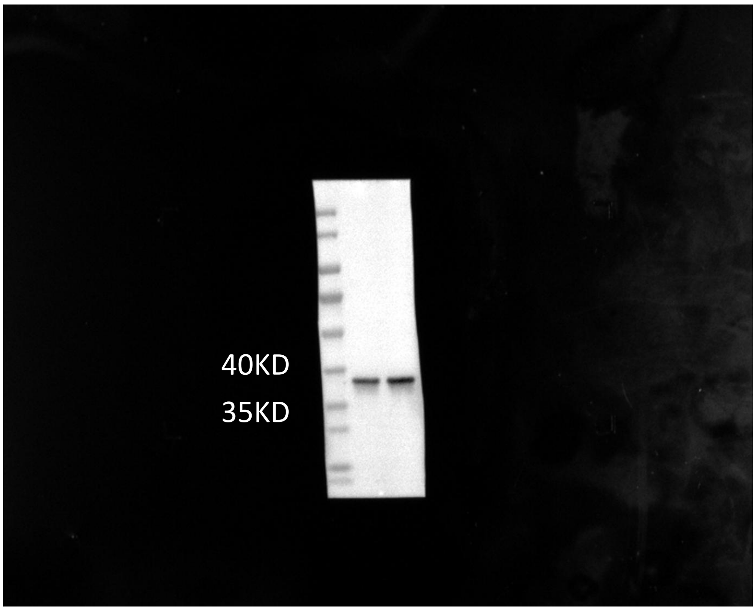

3E 1650 CGAS

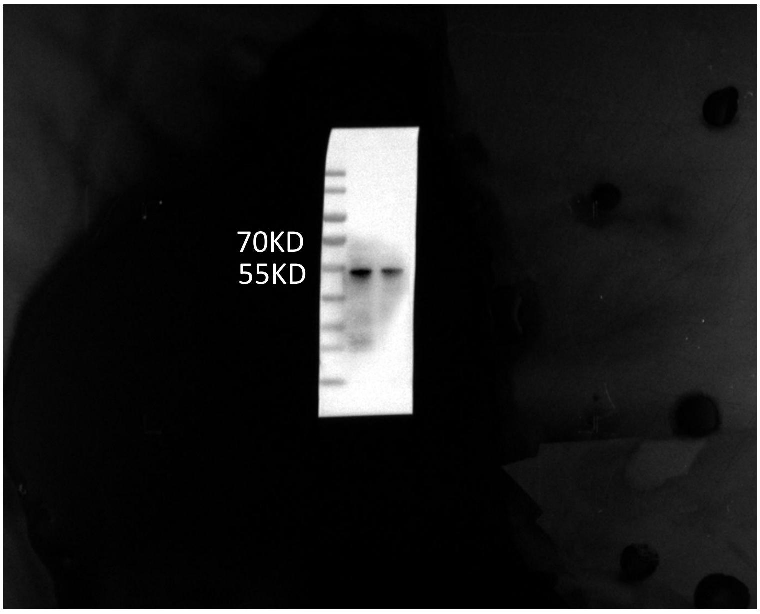

3E 1650 SING

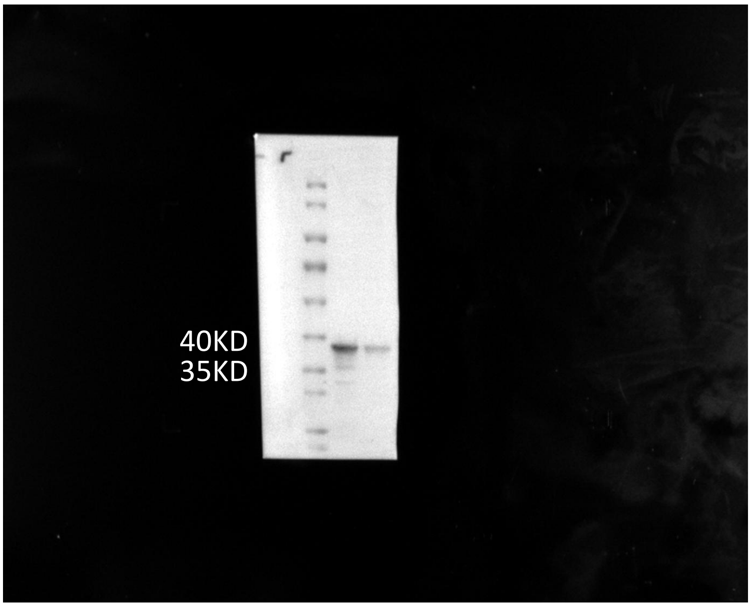

3E 1650 GAPDH

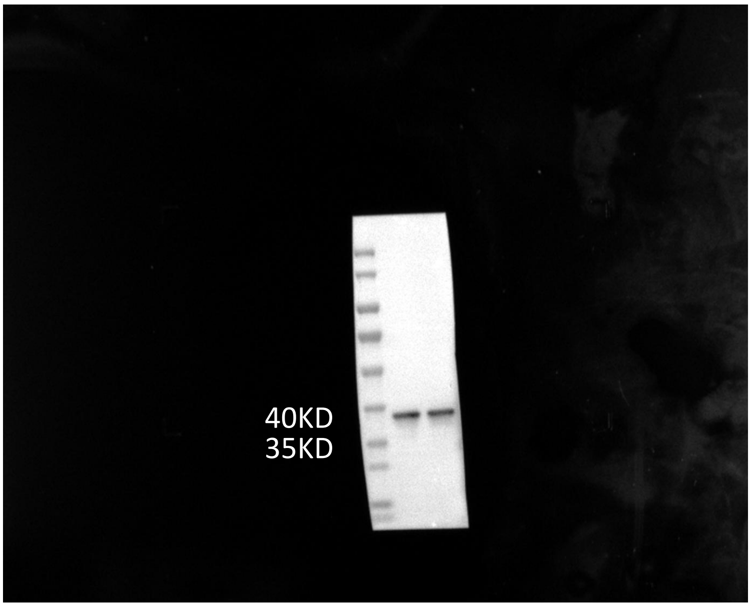

3E 1975 CGAS

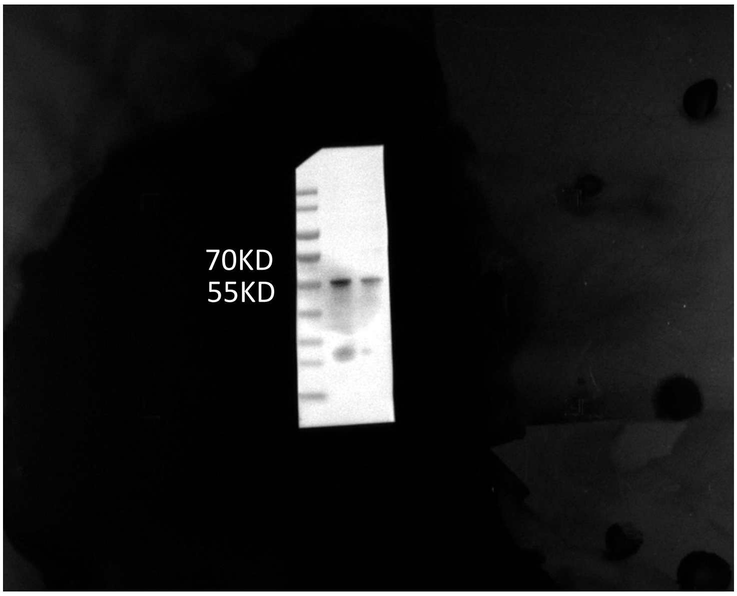

3E 1975 STING

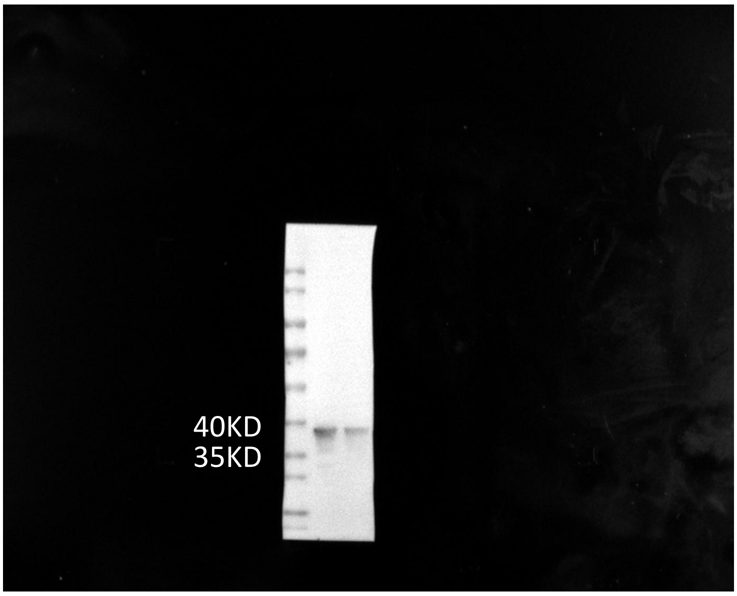

3E 1975 GAPDH

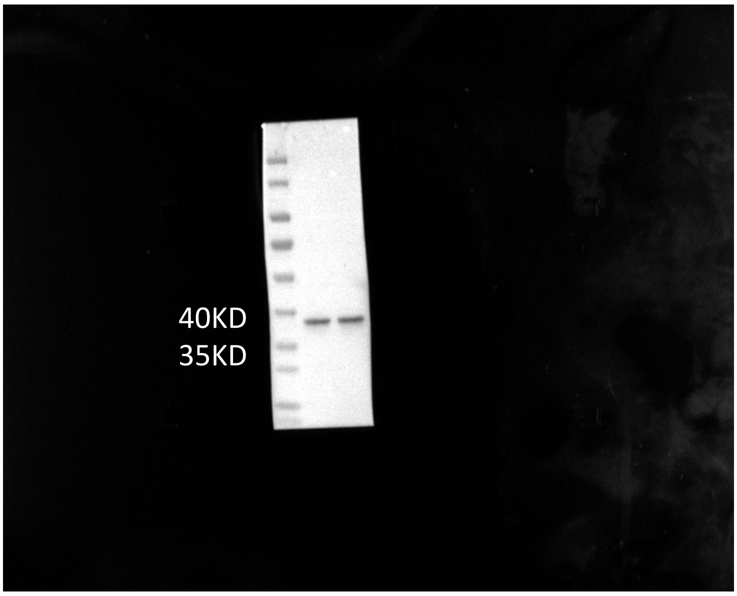

3F 1650 CGAS

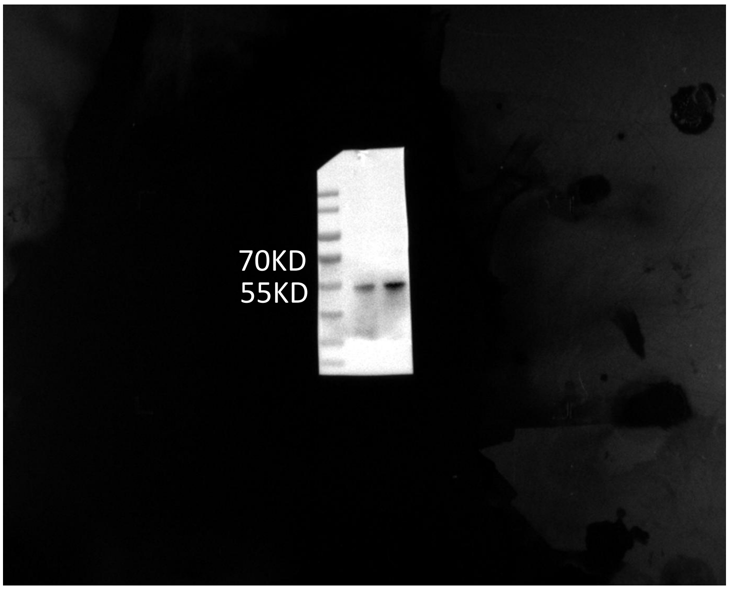

3F 1650 STING

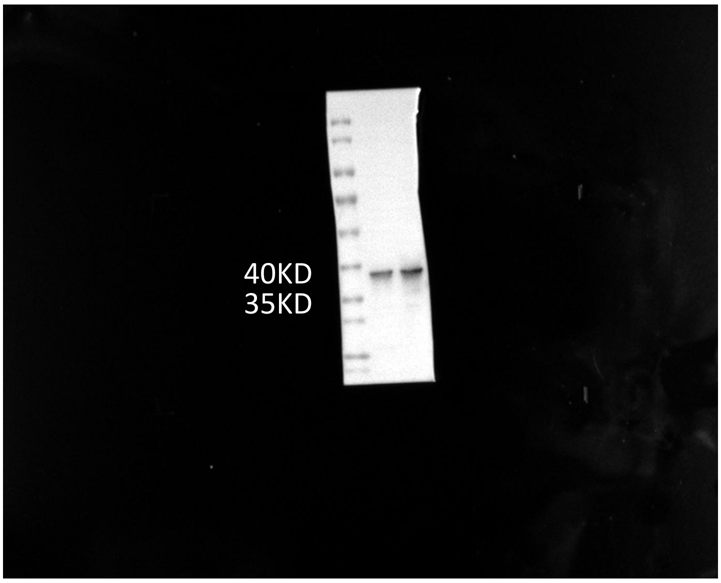

3F 1650 GAPDH

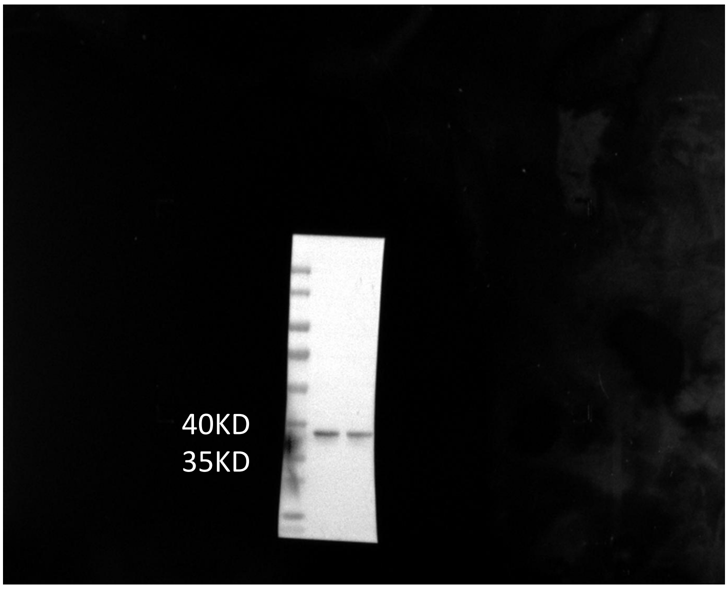

3F 1975 CGAS

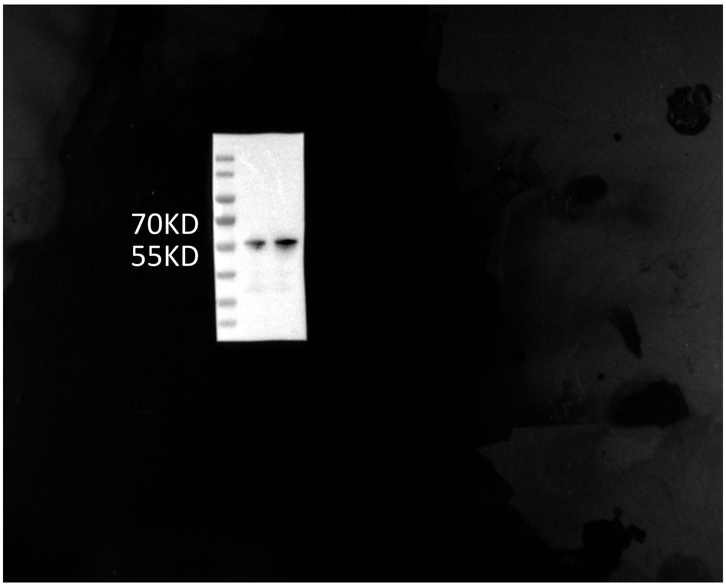

3F 1975 STING

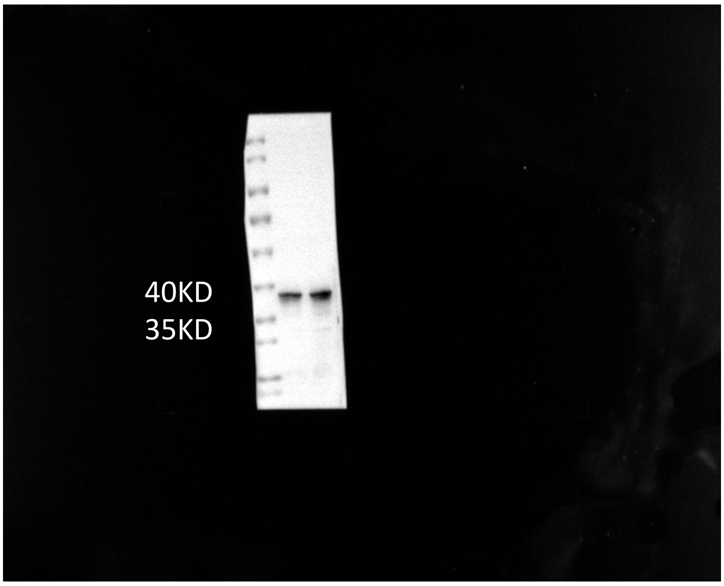

3F 1975 GAPDH

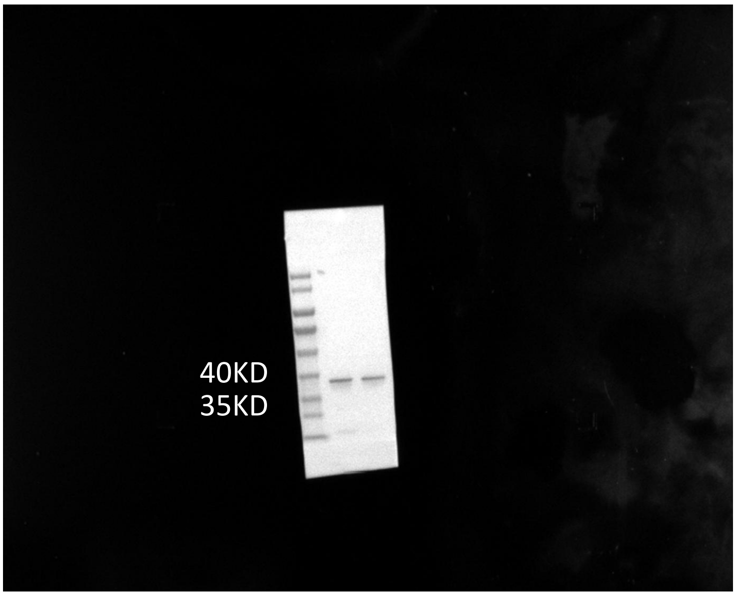

4F 1650 ZEB1

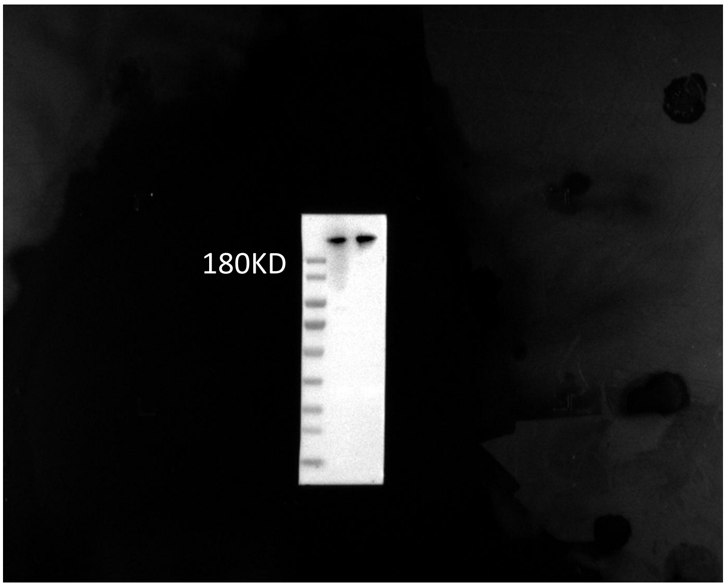

4F 1650 GAPDH

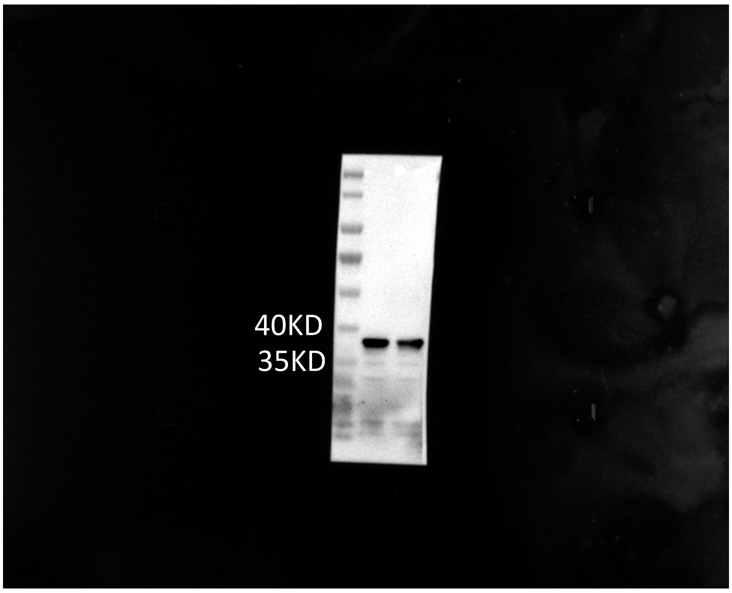

4F 1975 ZEB1

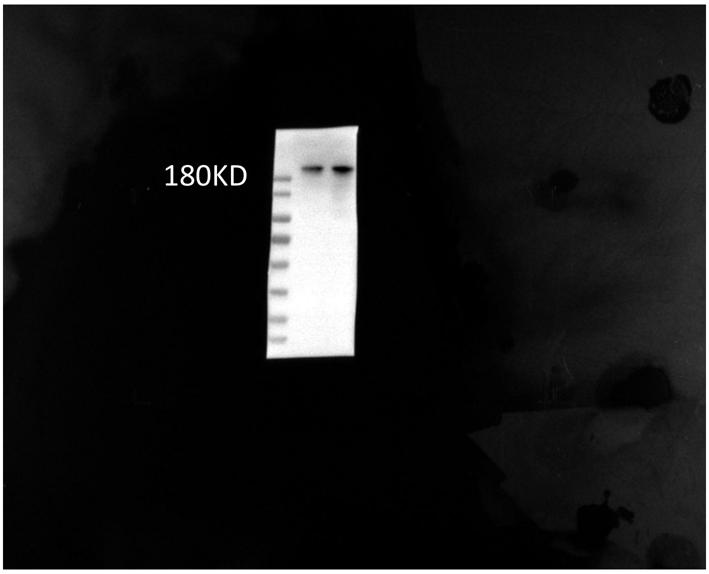

4F 1975 GAPDH

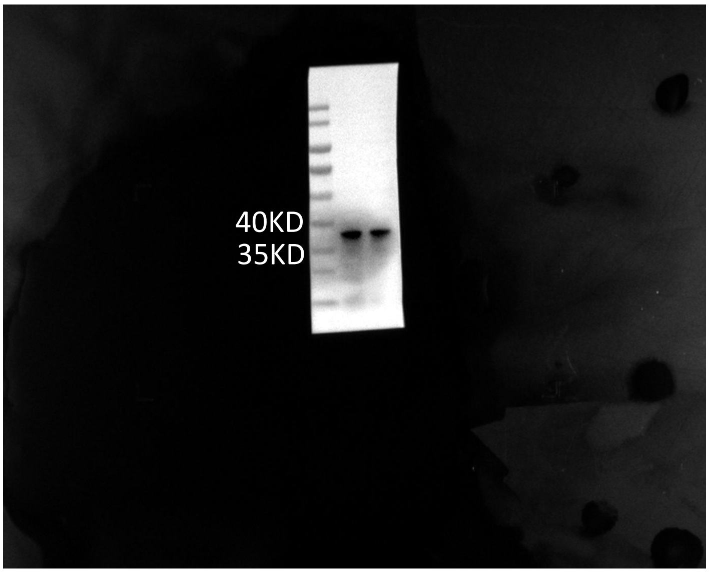

4H 1650 ZEB1

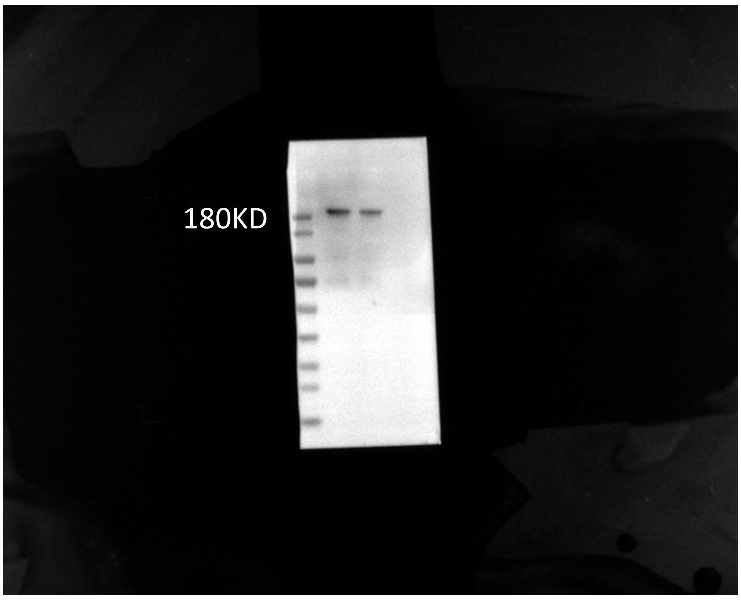

4H 1650 GAPDH

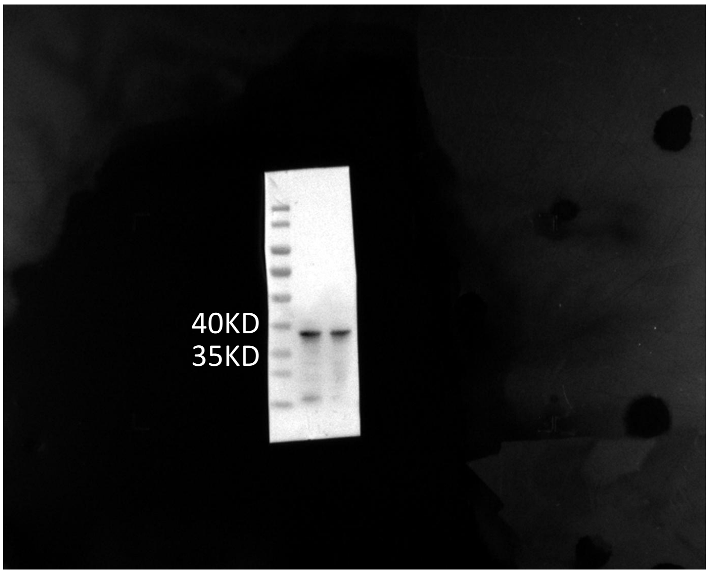

4H 1975 ZEB1

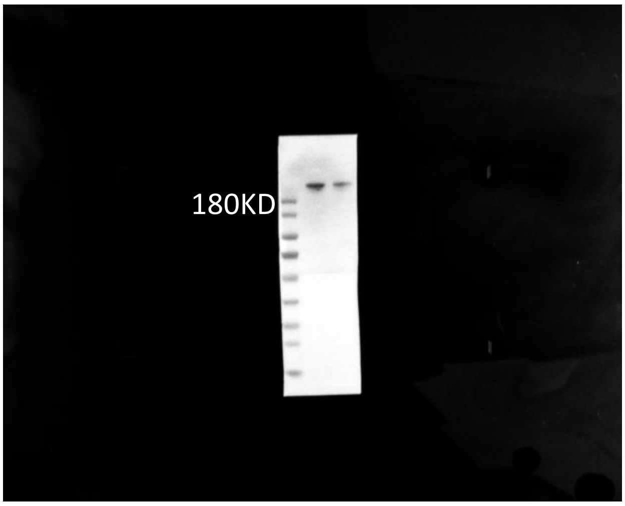

4H 1975 GAPDH

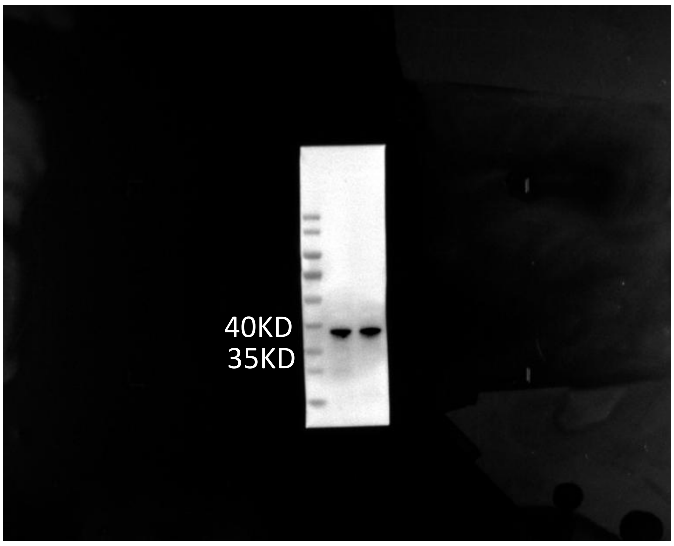

5A 1650 LC3B

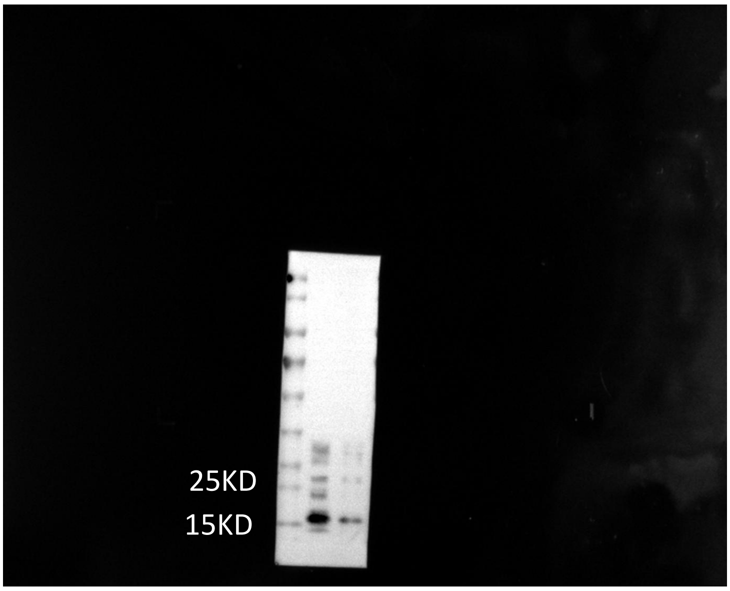

5A 1650 P62

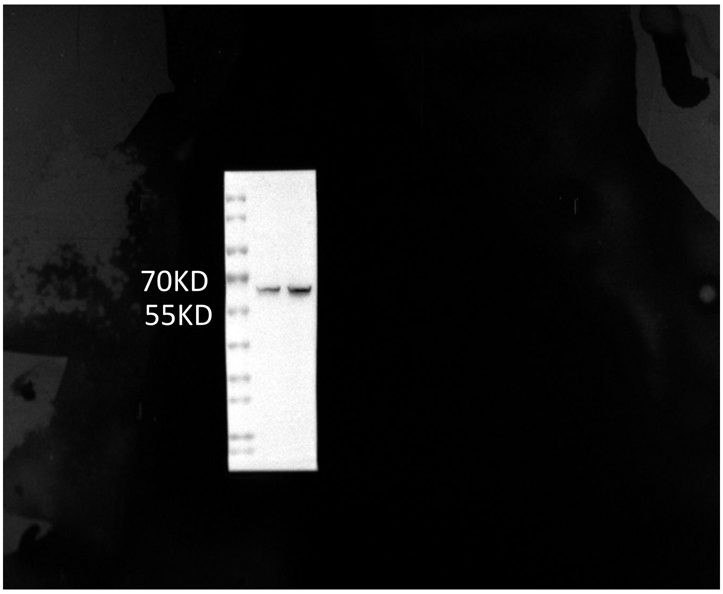

5A 1650 PINK1

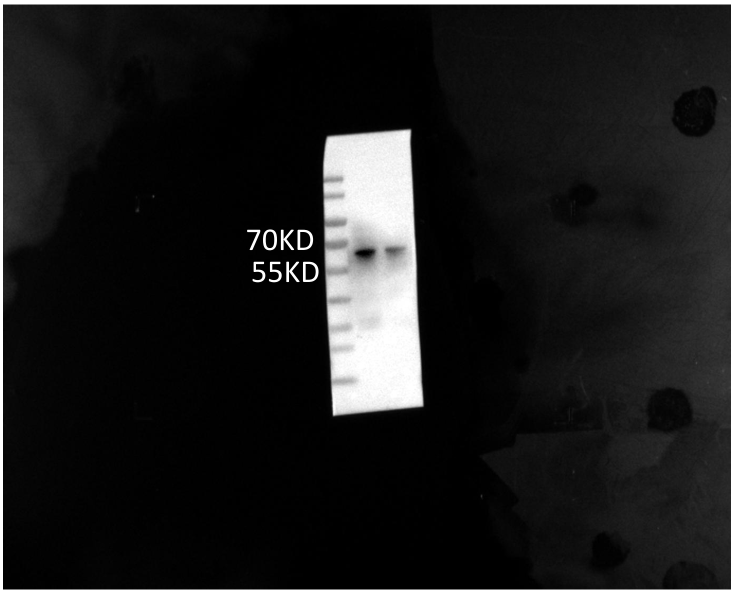

5A 1650 GAPDH

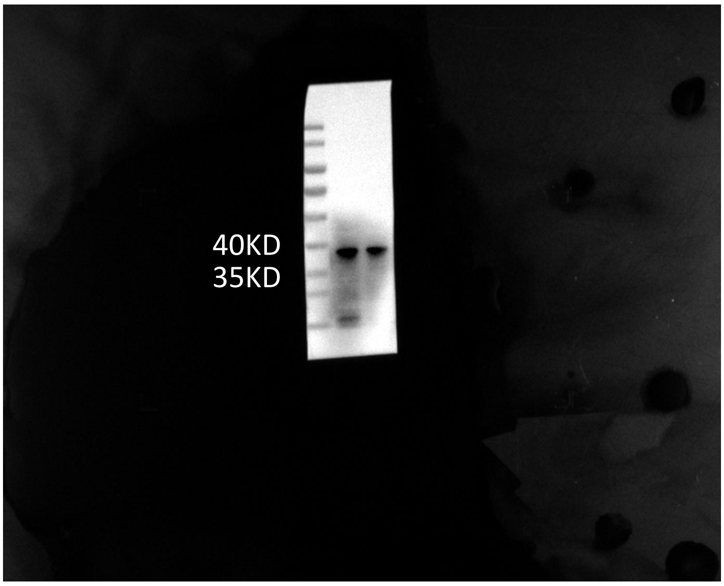

5A 1975 LC3B

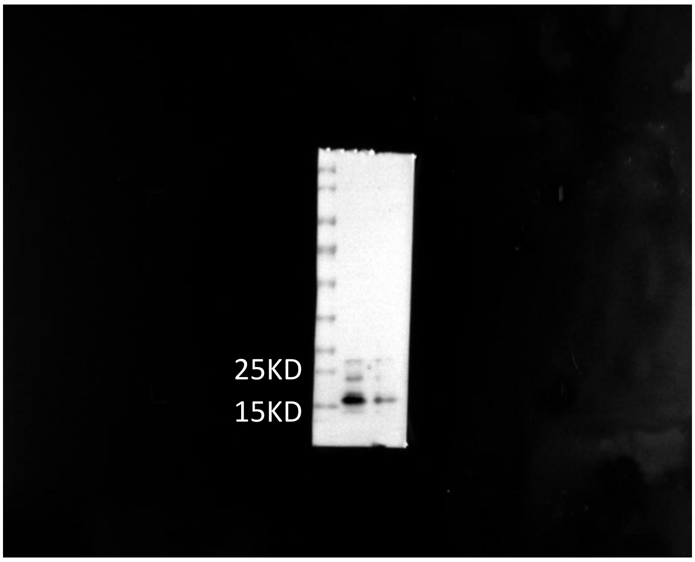

5A 1975 P62

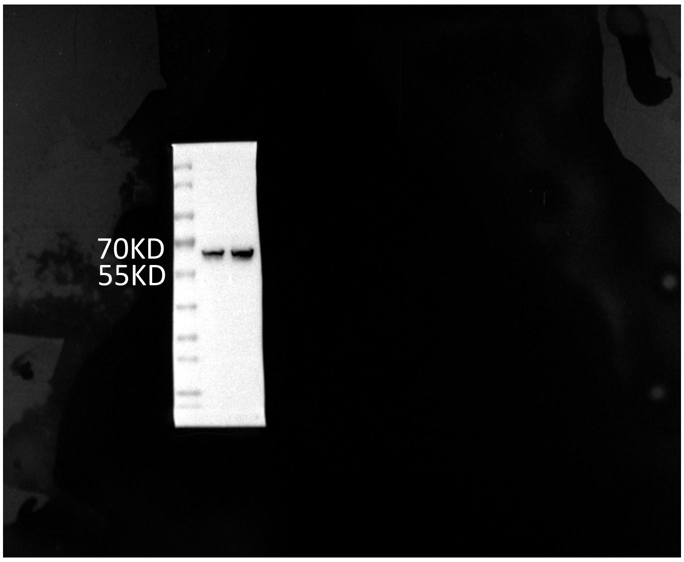

5A 1975 PINK1

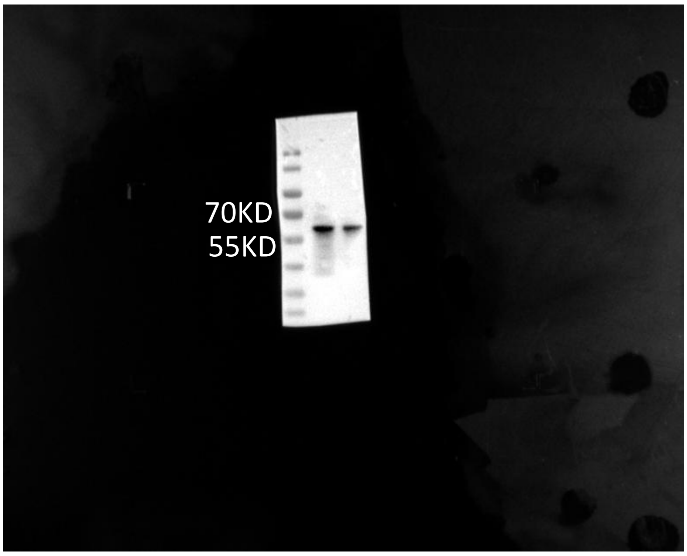

5A 1975 GAPDH

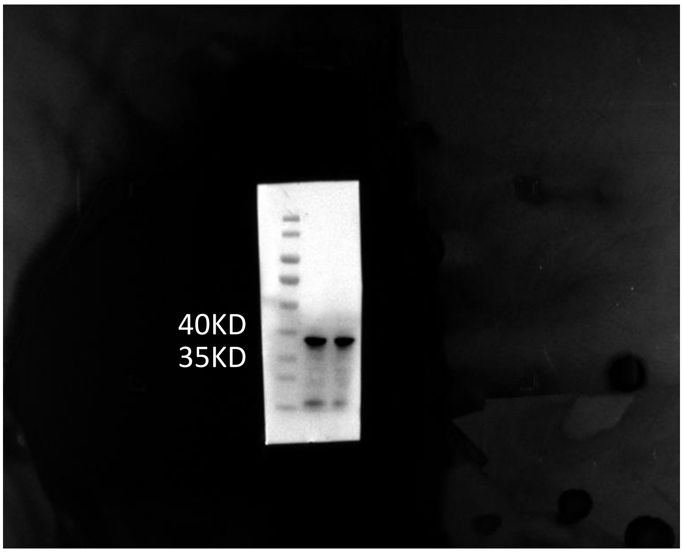

5E 1650 CGAS

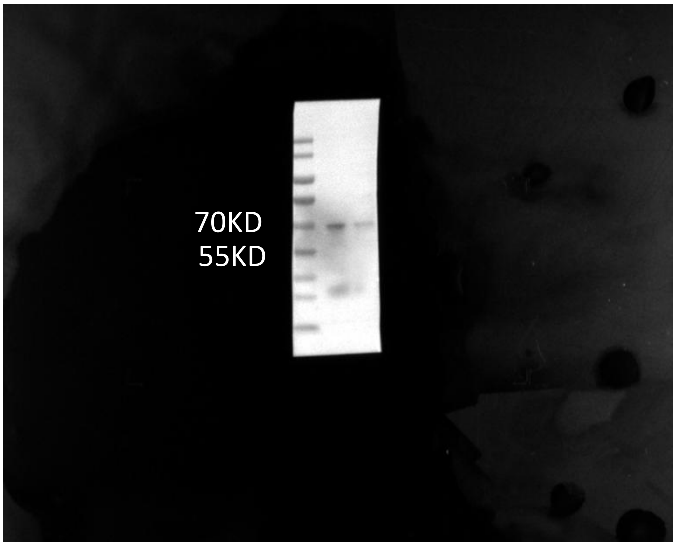

5E 1650 STING

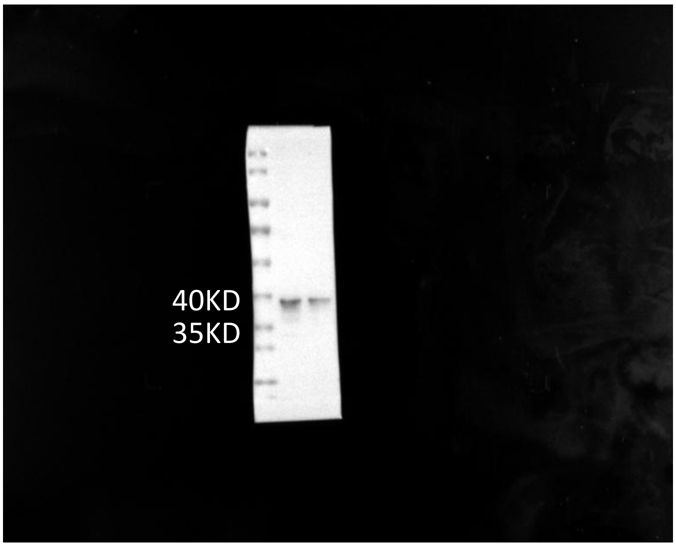

5E 1650 GAPDH

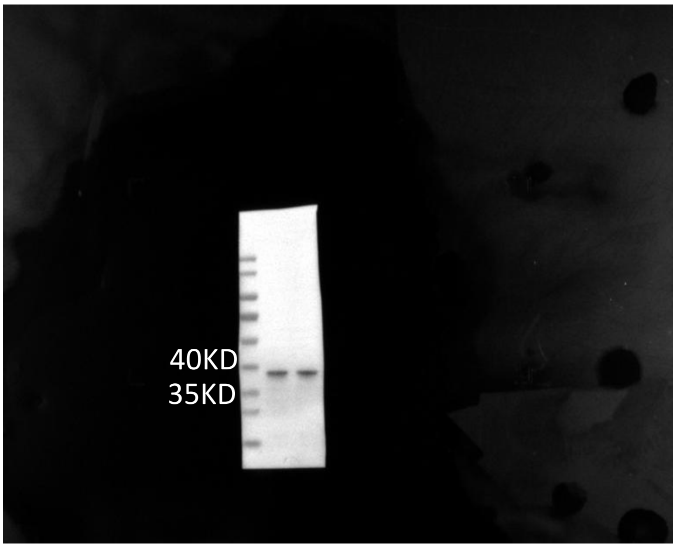

5E 1975 CGAS

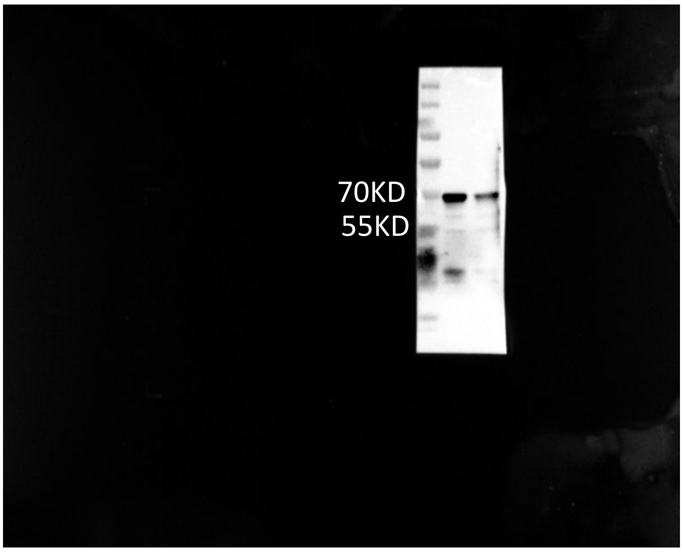

5E 1975 STING

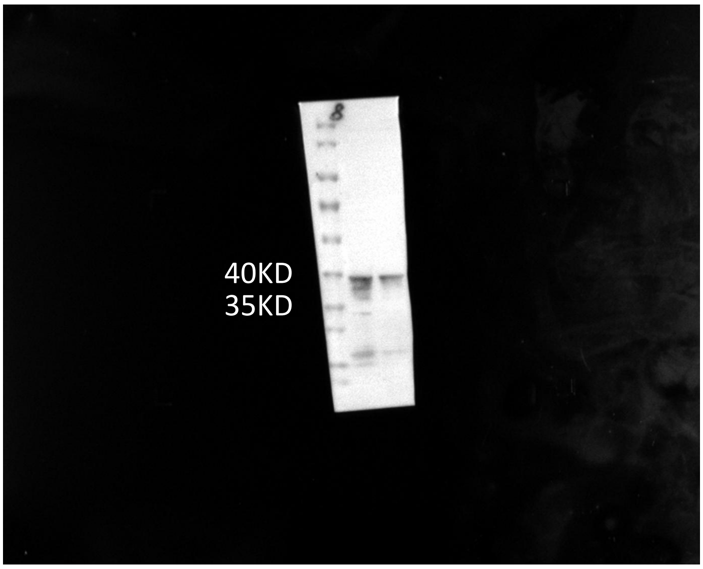

5E 1975 GAPDH

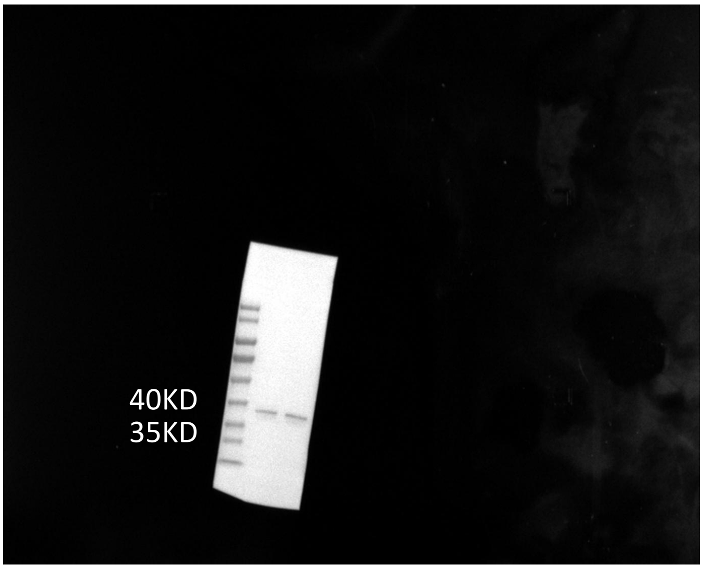

S2C 1975 CGAS

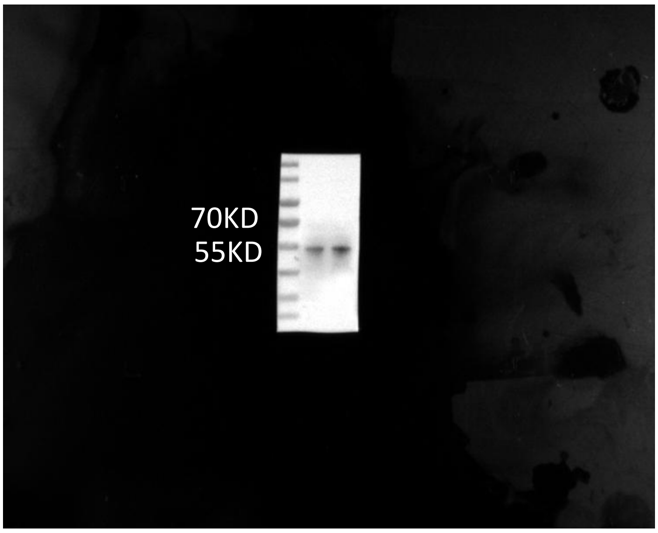

S2C 1975 STING

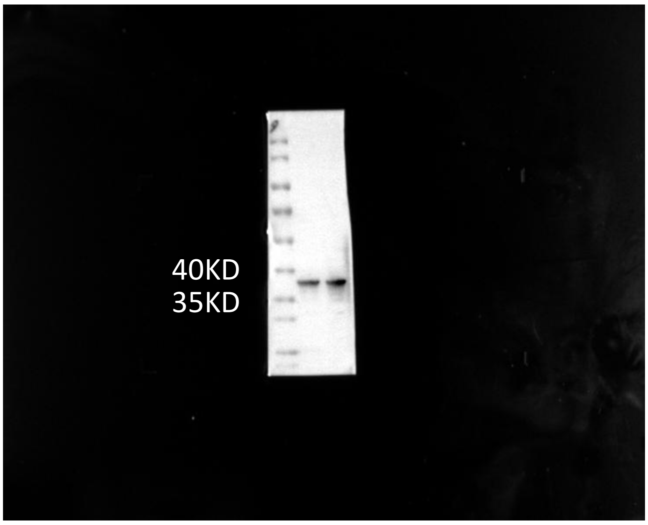

S2C 1975 GAPDH

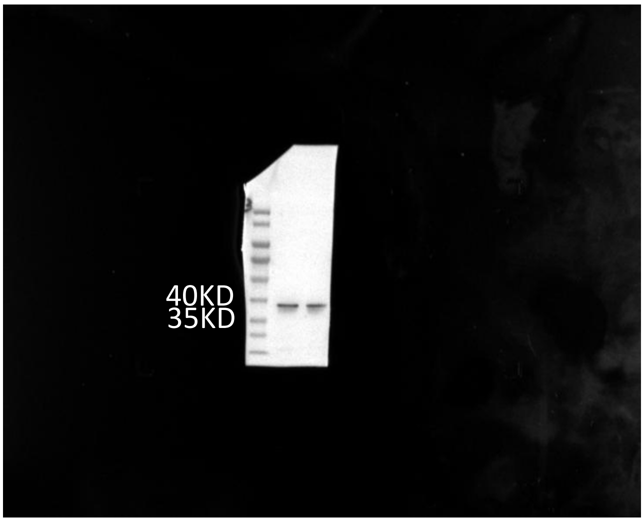

S2D 1975 STING

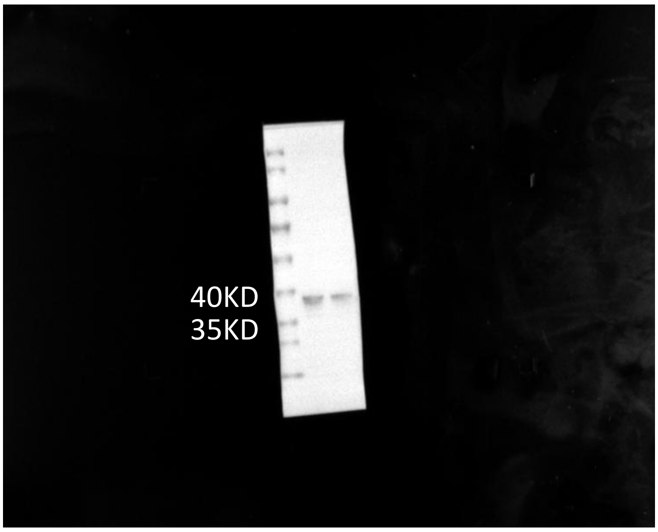

S2D 1975 GAPDH

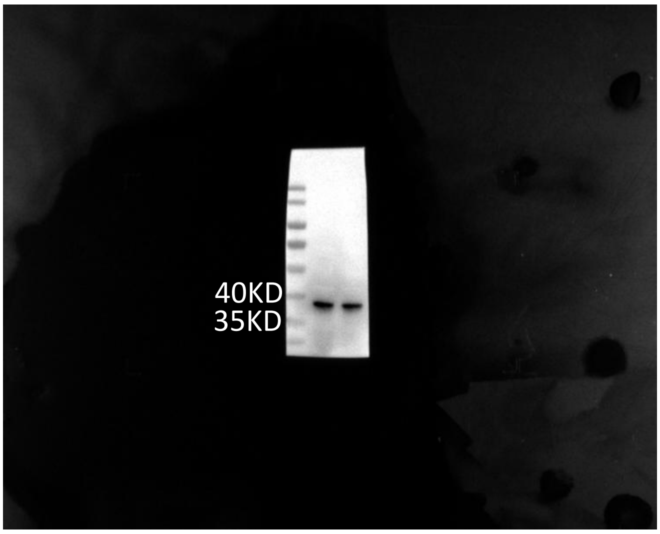
40KD
35KD
