# Supplementary material for: Residual Lung Cancer After Incomplete Microwave Ablation Exhibits cGAS–STING–ZEB1-Driven Malignant Progression
Source: Cancers (Basel). 2026 Aug 12;18(16):2594. doi: 10.3390/cancers18162594 (PMC13511148; doi:10.3390/cancers18162594)
Supplement: Supplementary file 1 [file cancers-18-02594-s001.zip › supplementary figures.pdf]

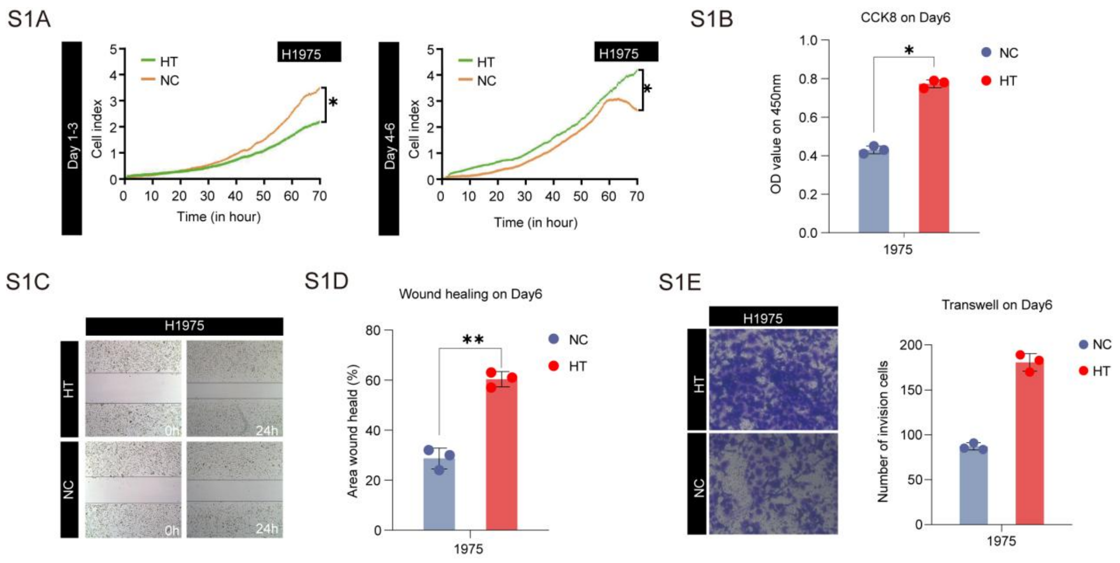

Figure S1

(S1A) Real-time cell analysis (RTCA) showing the proliferation dynamics of H1975 cells in the normal control (NC) and heat-treated (HT) groups. Cell index was continuously monitored for Day 1–3 (left) and Day 4–6 (right).

(S1B) Cell viability of H1975 cells measured by CCK-8 assay on Day 6. Optical density (OD) was recorded at 450 nm.

(S1C) Representative images of wound healing assays in H1975 cells at 0 h and 24 h after scratching in the NC and HT groups.

(S1D) Quantitative analysis of wound closure in H1975 cells on Day 6, expressed as the percentage of healed wound area.

(S1E) Transwell invasion assay of H1975 cells. Representative images (left) and quantitative analysis of the number of invaded cells (right) on Day 6. Scale bar: 50 µm.

Data are presented as mean ± SEM. *P < 0.05, **P < 0.01.

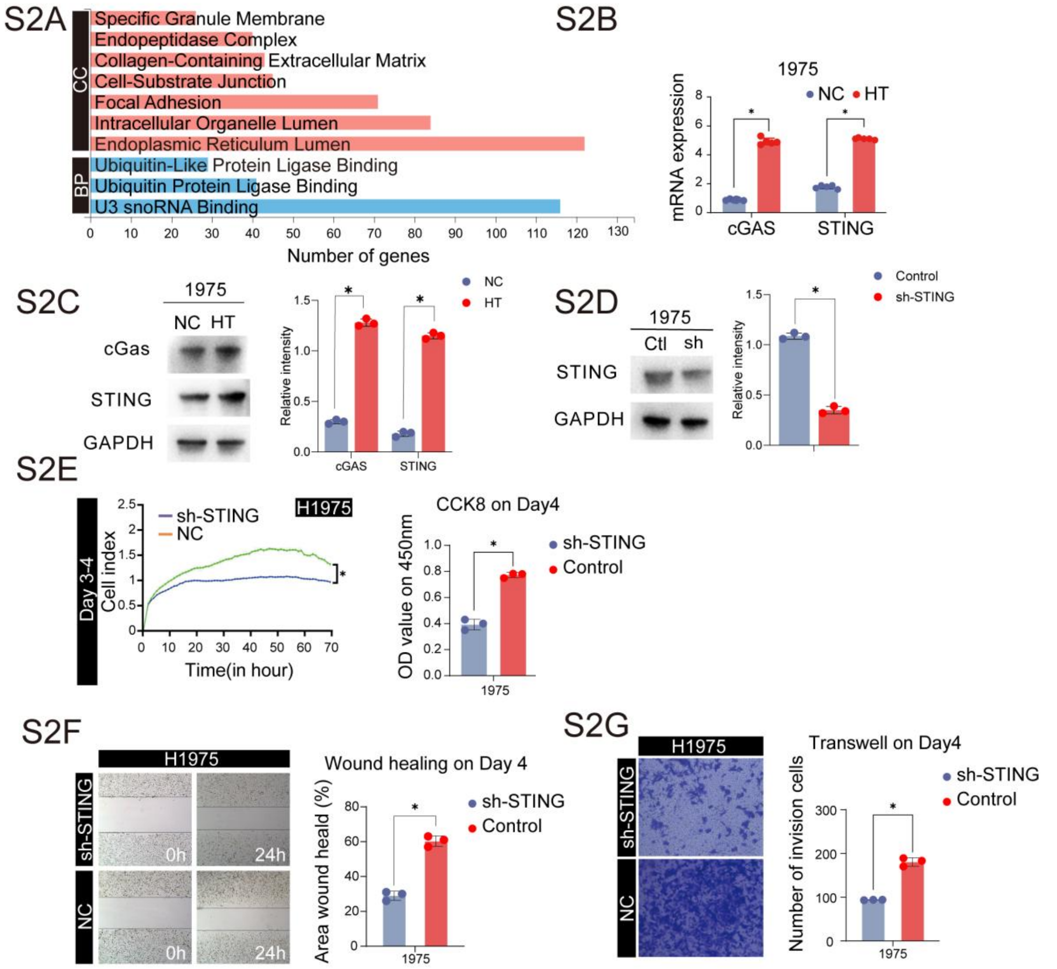

Figure S2

(S2A) GO enrichment analysis of differentially expressed genes (DEGs) between heat-treated (HT) and normal control (NC) groups, highlighting enriched cellular components and biological processes related to extracellular matrix organization and ubiquitin-related

functions.

(S2B) qRT-PCR analysis of cGAS and STING mRNA expression in H1975 cells following heat treatment (HT) compared with NC cells.

(S2C) Western blot analysis of cGAS and STING protein levels in NC and HT H1975 cells (left), with quantitative analysis of relative protein expression (right). GAPDH serves as the loading control.

(S2D) Western blot analysis validating STING knockdown efficiency in Control and sh-STING H1975 cells (left), with quantitative analysis shown on the right.

(S2E) Cell proliferation analysis of Control and sh-STING H1975 cells using RTCA (left), and cell viability measured by CCK-8 assay on Day 4 (right).

(S2F) Wound healing assay in Control and sh-STING H1975 cells. Representative images at 0 h and 24 h (left) and quantitative analysis of wound healed area on Day 4 (right).

(S2G) Transwell invasion assay performed on Day 4 in Control and sh-STING H1975 cells. Representative images (left) and quantitative analysis of invaded cells (right).

Data are presented as mean ± SEM. *$P < 0.05$.

Abbreviations: HT, heat-treated; NC, normal control; GO, Gene Ontology; DEG, differentially expressed gene; qRT-PCR, quantitative real-time polymerase chain reaction; cGAS, cyclic GMP-AMP synthase; STING, stimulator of interferon genes; sh, short hairpin RNA; RTCA, real-time cell analysis; CCK-8, Cell Counting Kit-8.
